# Integrative spatially resolved proteomic and metabolomic imaging reveals synovitis endotypes implicated in osteoarthritis progression

**DOI:** 10.7150/thno.117788

**Published:** 2025-08-30

**Authors:** Lin Zhu, Xin Diao, Chenrui Yuan, Chun Man Lawrence Lau, Jianing Wang, Wenlong Wu, Ali Mobasheri, Xavier Houard, Chunyi Wen, Zongwei Cai

**Affiliations:** 1 State Key Laboratory of Environmental and Biological Analysis, Department of Chemistry, Hong Kong Baptist University, Hong Kong SAR, 999077, China; 2 Department of Orthopaedics and Traumatology, School of Clinical Medicine, Li Ka Shing Faculty of Medicine, The University of Hong Kong, Pokfulam, HKSAR, China; 3 Research Unit of Medical Imaging, Physics and Technology, Faculty of Medicine, University of Oulu, PO Box 5000, FI-90014 Oulu, Finland; 4 Department of Regenerative Medicine, State Research Institute Centre for Innovative Medicine, Vilnius, Lithuania; 5 Department of Joint Surgery, First Affiliated Hospital of Sun Yat-sen University, Guangzhou, Guangdong, China; 6 Faculty of Medicine, Université de Liège, Liège, Belgium; 7 Sorbonne Université, INSERM, Centre de Recherche Saint-Antoine, CRSA, F-75012, Paris, France; 8 Department of Biomedical Engineering, The Hong Kong Polytechnic University, Hung Hom, Hong Kong, China; 9 Eastern Institute of Technology, Ningbo, 315200, China

**Keywords:** osteoarthritis, synovitis, molecular endotype, spatial proteomics, MALDI-MSI

## Abstract

**Rationale**: Synovial fibrosis, driven by myofibroblast activation and extracellular matrix remodelling, is fundamental in osteoarthritis (OA) pathogenesis but remains poorly understood due to the spatial heterogeneity of synovial inflammation (synovitis). Accurate molecular endotyping of synovial inflammation is essential for effective treatment of OA given its multifactorial nature, yet it requires integrating multiple layers of information with spatial context due to the significant heterogeneity of the tissue.

**Methods:** In this proof-of-concept study, we leveraged MALDI mass spectrometry imaging to achieve spatial metabolomic maps that complement high-content proteomic profiles. Microflow liquid chromatography was employed to improve the robustness and throughput of spatial proteomics. By coupling these spatially resolved datasets, we establish a pseudo time trajectory of heterogeneous synovitis in human knee OA using an integrative framework of spatially resolved proteomics and matrix-assisted laser desorption/ionization mass spectrometry imaging.

**Results**: Clustering 3534 proteins and 79 energy metabolites from spatial proteomic and metabolomic image datasets reveals four distinct functional stages of OA synovitis, i.e., quiescent, microvasculopathic, pre-fibrotic, and post-fibrotic stages, which enables construction of a corresponding pseudo time. Network analyses elucidate the functional links among these stages, highlighting an immune-metabolic axis from endothelial injury and microvascular thrombosis toward myofibroblast activation.

**Conclusions:** This integrative multi-omics imaging approach informs the inflammatory endotype of OA, supporting a vascular aetiology of synovial fibrosis and offering mechanistic insights that could inform more targeted therapeutic strategies. Validation in larger, stratified patient cohorts will be critical to refine our findings and accelerate their clinical usages.

## Introduction

Osteoarthritis (OA) is a leading degenerative disease in synovial joints, affecting over 500 million people worldwide^1^. Synovial inflammation significantly contributes to OA's radiographic and symptomatic progression^2^, ^3^. The synovium consists of an intimal layer rich in macrophages and fibroblasts, and a subintimal layer containing loose connective tissue, blood vessels, and various immune cells^4^. Complex cell-cell interactions within synovial layers produce diverse inflammatory phenotypes, creating substantial heterogeneity in OA synovitis^5^. The variability undermines our understanding of OA pathogenesis and potentially hampers the development of effective targeted therapies^6^.

There is a growing need for spatially resolved technologies that capture the complex pathobiology of synovium, prompting a surge of interest in novel approaches^7^, ^8^. Recent work combining spatial transcriptomics with multiplex protein profiling, targeting 132 molecules, has begun to clarify the role of synovial innate immunity in knee OA pain^9^. However, immunoassay-based methods, while powerful, are inherently limited by antibody availability, cost, and their limited number of targeting antigens, thereby restricting deep, unbiased proteome profiling^10^. In contrast, laser capture microdissection (LCM)-coupled proteomics can provide untargeted, spatially resolved datasets from single cells and tissue microenvironments^11^-^14^. By sampling in a raster grid, 2,000 to 4,000 proteins can be imaged in a single tissue section^14^, ^15^. Yet, this method remains challenging for synovial tissue due to lengthy liquid chromatography (LC) protocols requiring hundreds of injections, each often exceeding 45 minutes per individual injection^4^, ^10^.

Environmental and nutritional factors can each trigger or exacerbate synovitis through distinct mechanisms^4^-^6^, ^16^, making single-omics approaches insufficient for comprehensive molecular endotyping. Because protein-metabolite interactions largely govern tissue function, an untargeted, in-depth method that integrates proteomics and metabolomics with histopathological information is essential to define the molecular endotypes of OA^17^, ^18^. Matrix-assisted laser desorption/ionization-mass spectrometry imaging (MALDI-MSI) uniquely provides label-free, unbiased metabolic profiling of tissue sections^19^-^21^, but it remains underutilized for energy metabolite analysis in synovium. Although workflows combining MALDI-MSI and laser capture microdissection (LCM)-based proteomics offer a potential solution^22^-^24^, the low throughput of proteomics continues to pose a major obstacle.

In this proof-of-concept study, we demonstrate an integrative framework of spatially resolved proteomics and MALDI-MSI in human knee OA synovium. By adopting micro-flow LC, we substantially reduced proteomics run times, enabling seamless integration with imaging and histology. This approach captures key vascular dysfunction, thrombotic, and fibrotic processes and outlines a pseudotime trajectory of OA synovitis. Ultimately, our integrated strategy may facilitate personalized treatment and patient stratification based on individual molecular endotypes, laying the groundwork for future, large-scale cohort studies.

## Results

### General overview and strategic design of the study

**Figure 1:**
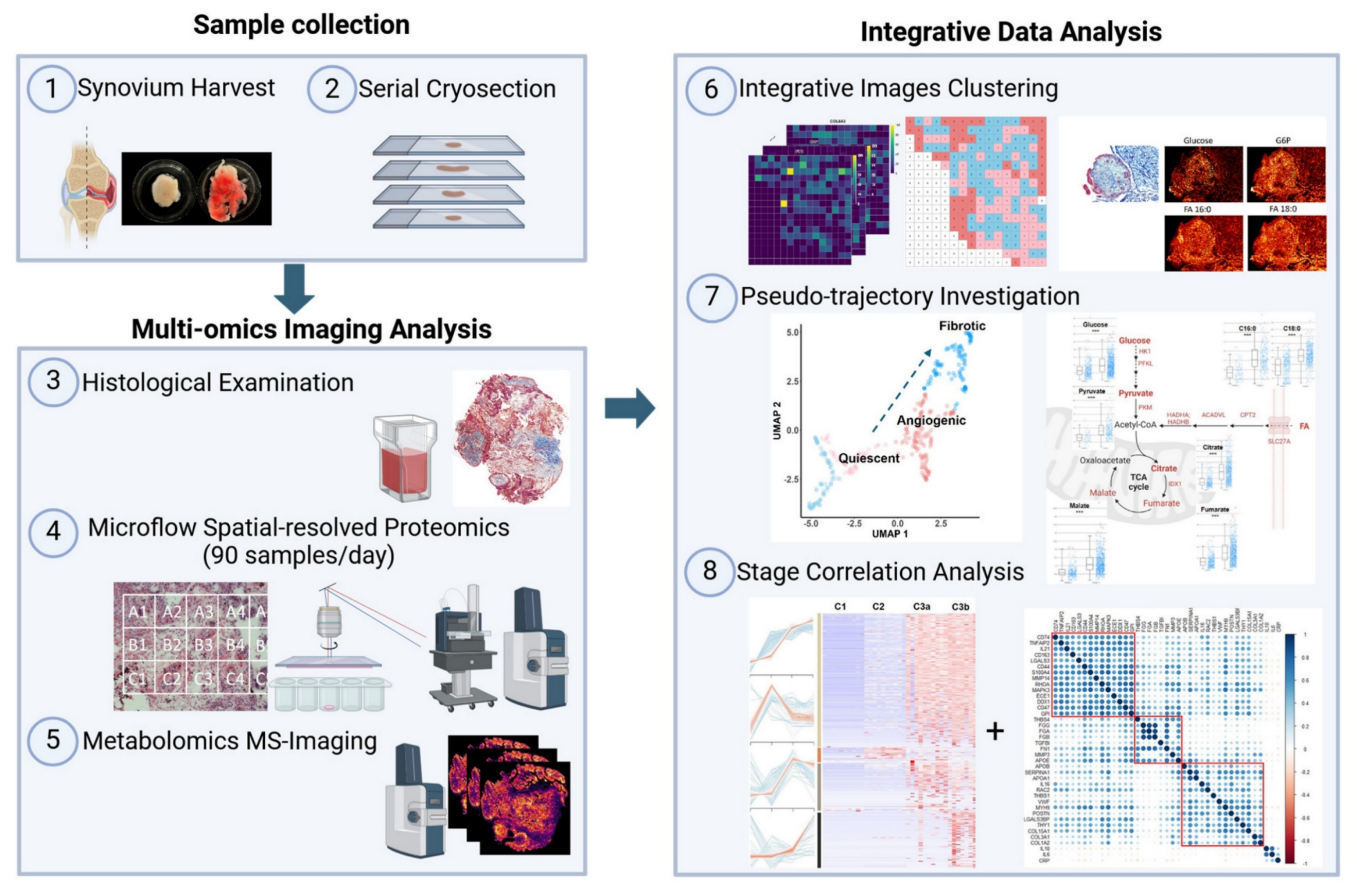
** MS-based multi-omics imaging strategy for OA synovium endotyping**.

We established an integrated multi-omics imaging strategy composed of histological, metabolomics, and proteomics images to effectively characterize and categorize the heterogeneous microregions (“niches”) within OA synovium (**Figure 1**). Two representative synovial samples from a single patient undergoing total knee replacement were harvested post macroscopic examination, followed by serial cryosection and morphological characterization using Masson's trichrome staining (**Figure S1**). Untargeted heatmaps of thousands of proteins were then obtained in a raster grid. A micro-flow strategy coupling the use of a 5 cm long C18 UHPLC analytic column was applied, which identified 4400-4800 protein groups depending on LC running time (**Figure S2**). Based on the LC optimization results, we applied the 15-min LC gradient for actual sample analysis, enabling the analysis of ~90 samples per day. MALDI-MSI was performed on a consecutive slide, focusing on energy metabolites that provide tissue functional status. Subsequent integrative analyses were performed to enable accurate molecular endotyping for OA.

### Distinct spatial heterogeneity was observed in OA knee synovium via multimodal imaging

**Figure 2:**
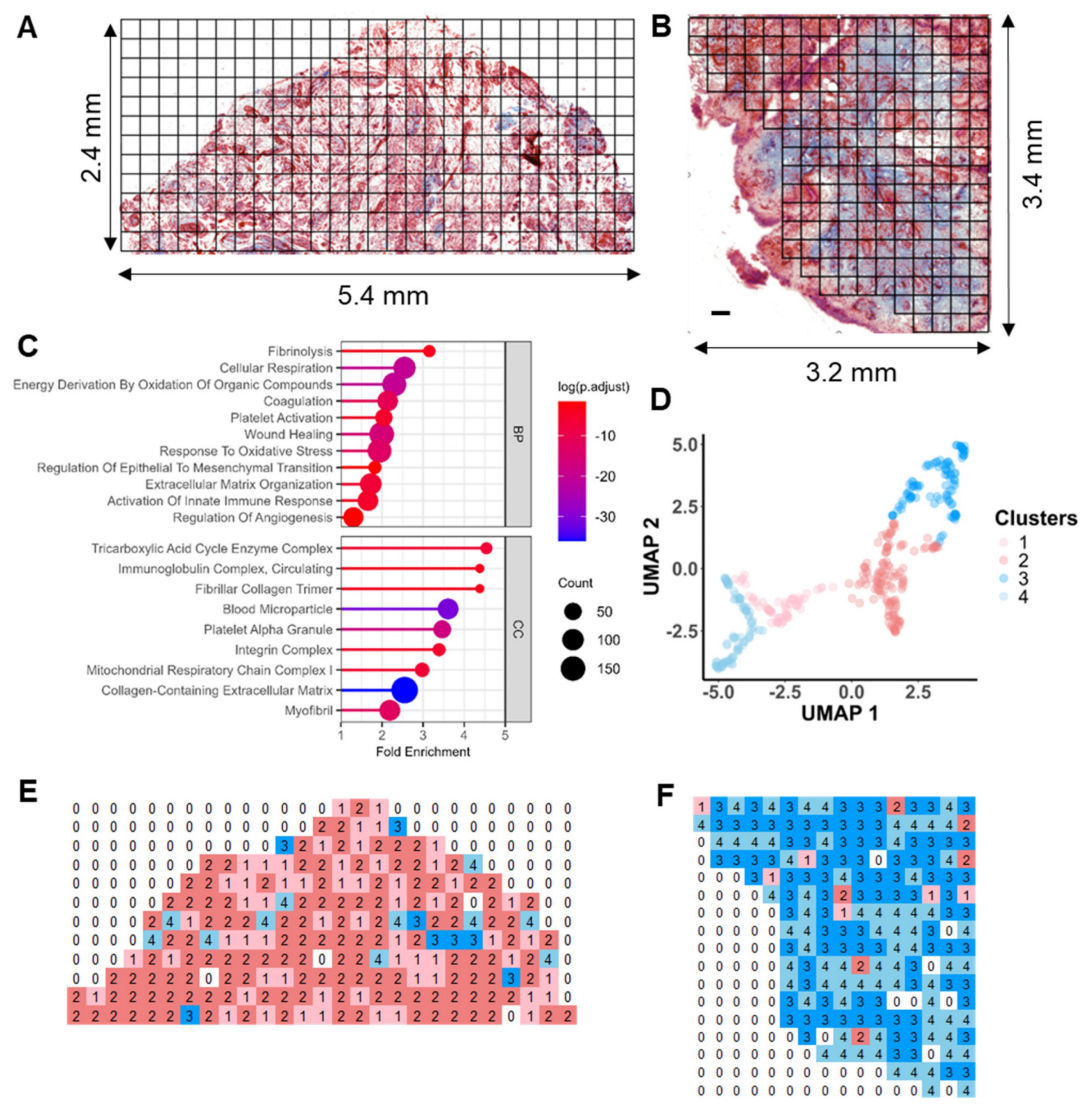
** Distinct spatial heterogeneity of protein expression identified in OA synovium. A-B** LCM sampling grid for two selected representative regions with histological imaging reference. The samples were Masson Trichrome stained. Scale bar 200 uM. **C** Selective GO enrichment terms of proteins identified in the study, relevant to OA onset or pathogenesis; BP biological pathways; CC cellular components. **D** Two-dimensional (2D) UMAP projection for all 390 voxel samples generated using k-means clustering. Four clusters were identified and are represented by different colours. Each dot represents a single voxel. **E-F** Stand-alone spatial mapping of cluster assignments of two distinct regions.

As proof of concept, we selected regions of the same synovium, each showing different degrees of fibrosis and angiogenesis, with a total surface area of 16.2 mm^2^ for subsequent spatial proteomics analysis at 200 µm resolution (**Figure 2A-B**). This generated 390 voxel samples and 3534 quantified proteins within 100 hours of analytic time. A panel of markers related to OA or synovitis were found, including interleukins (IL-6, IL-1β, IL18, etc.), damage-associated molecular pattern (DAMP) markers such as S100, MMP, and heat shock (HSP) family proteins, as well as reactive oxygen species (ROS) or metabolic regulators (**Figure S3A**), which covering a wide range of biological functions and pathways related to OA onset or pathogenesis (**Figure 2C**). The summed and mean intensity per voxel were consistent across both ROIs (**Figure S3**), allowing us to compare inter-slide quantitatively for clustering analysis of voxels.

After filtering and normalization, all sample data were projected onto a 2-dimensional space via UMAP (2D-UMAP) for clustering without considering their spatial coordinates (**Figure 2D**). The voxels formed four clusters on UMAP, as indicated in different colours. We then mapped the four identified clusters with their spatial locations (**Figure 2E-F**) and generated overlapping images of histology and protein clusters (**Figure S3E-F**). As expected, proteomic clusters correlated with histology but did not completely overlap, representing an additional layer of information. OA functional markers heatmaps were plotted based on their intensity with spatial coordinates (**Figure S4**), covering fibrotic, myofibroblast activation (MFA), microvasculopathic, and thrombotic processes. In short, highly heterogeneous images were collected at morphological and proteomic levels, enabling the comprehensive characterization of OA functional niches.

### Pseudo-temporal trajectory analysis revealed typical OA-associated functional stages

**Figure 3:**
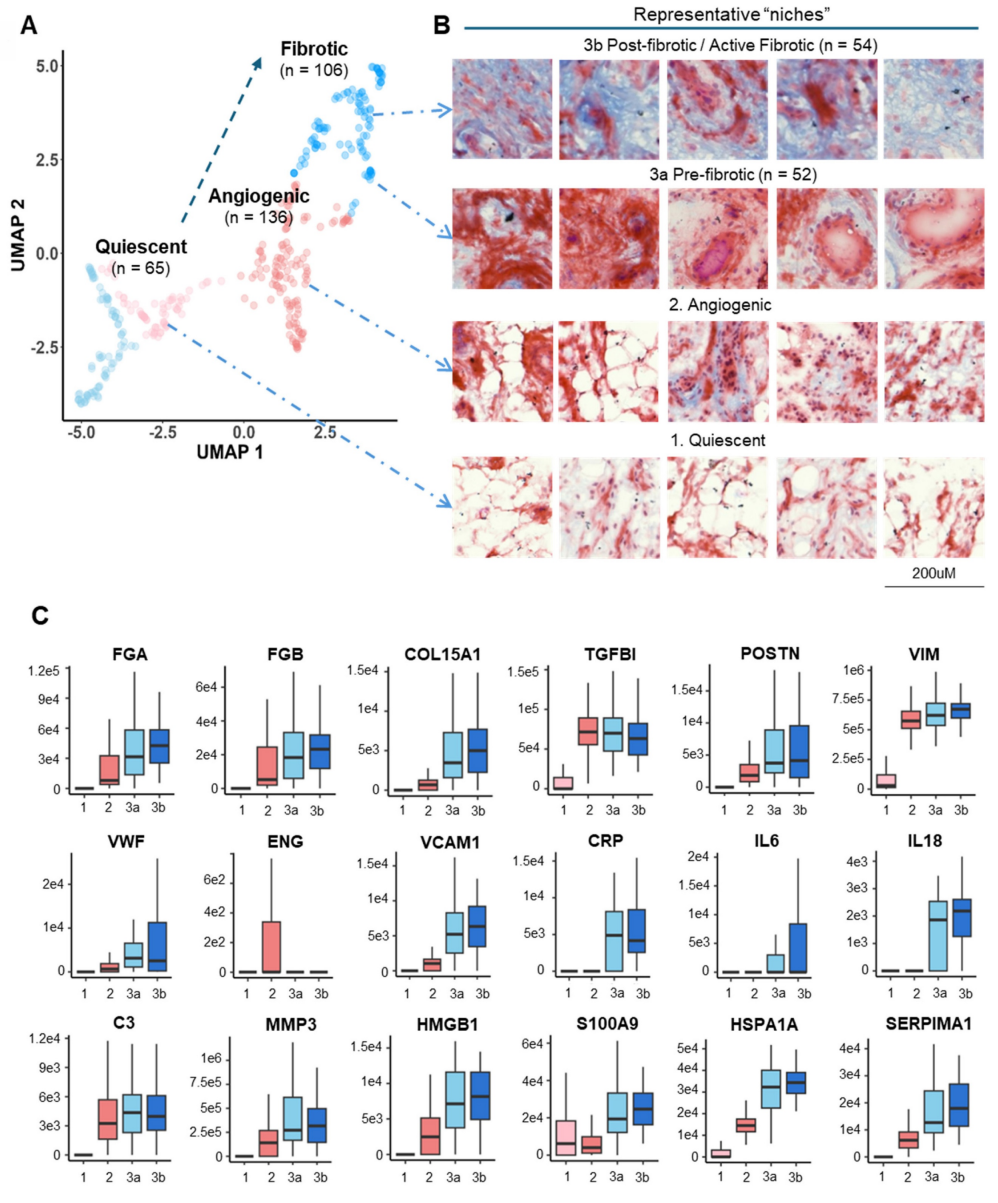
**Pseudo-time trajectory analysis revealed typical OA-associated functional stages. A** Functional stage identified by 2D-UMAP analysis of spatially resolved proteomic data. Stages were assigned based on protein expression profiles and subjective evaluation of corresponding histological staining. **B** Representative histological images corresponding to the identified functional stages. The proposed “Fibrotic” cluster was subdivided based on histological staining (blueness): 3a, Pre-fibrotic; 3b, Post/Active Fibrotic. **C** Boxplots of selected OA-related functional markers. The median and standard deviation of signal intensities are shown for all voxels within each functional stage. Abbreviations: FGA/FGB fibrinogen alpha/beta chain; COL15A1 Collagen Type XV Alpha 1 Chain; TGFBI Transforming growth factor beta induced; VWF Von Willebrand Factor; ENG Endoglin; VIM Vimentin; VCAM1 Vascular cell adhesion protein 1; CRP C-Reactive Protein; IL6 Interleukin 6; IL18 Interleukin 18; C3 Complement C3; MMP3 Matrix Metallopeptidase 3; HMGB1 High Mobility Group Box 1; S100A9 S100 Calcium Binding Protein A9; HSPA1A Heat Shock Protein Family A (Hsp70) Member 1A; SERPINA1 Serpin Family A Member 1.

We further characterized and catalogued different clusters into functional stages using expression profiles of key OA markers in combination with histological staining evaluation (**Figure 3**). A set of representative staining images from each functional niche is shown in **Figure 3B**, with known OA-related markers for parallel stage assessment (**Figure 3C, S5**). Three functional clusters were instantly identified: Cluster 1 (Quiescent), which is metabolic silence (low expression of Pyruvate Dehydrogenase E1 Subunit Alpha 1, PDHA1) and little inflammatory cytokine (IL6 and CRP) expression, representing a relatively quiescent state of synovium. Cluster 2 (Microvasculopathic) is marked by elevated expression of two well-known markers, Von Willebrand factor (vWF) and Endoglin (ENG), which are indicative of endothelial and microvascular dysfunction. We also observed microvascular formation in corresponding regions via Masson's trichrome staining (**Figure 3B**), confirming active angiogenic events on-going in the microenvironments of cluster 2. Cluster 3 represents fibrotic tissue with the highest expression of collagens, indicated by both elevated expression of collagen and blue colour in histochemical staining. Accumulation of extracellular matrix remodeling activators such as MMP3 and HSPA1A, as well as myofibroblast markers (POSTN, VIM) further confirmed fibrotic stage (**Figure 3B**). A non-progressive Cluster 4 was characterized with an overall similar expression pattern of C1 but with elevated IL6 and IL10 levels, while no repairing process-related protein (such as TGF-β related proteins) was found in c4, and was therefore excluded from the trajectory analysis.

Intriguingly, when examining using histological information, we unexpectedly revealed two subclusters within cluster 3: cluster 3a (pre-fibrotic), stained red and associated with blood vessels, and cluster 3b (post-fibrotic or active fibrotic), generally stained blue, indicative of high collagen content (**Figure 3B**). This observation indicated that cluster 3a may represent a transitional stage where microvasculopathy and fibrosis co-exist and interplay, demonstrating a pseudo-temporal trajectory of OA development (**Figure 3A**) and encouraging us to explore the underlying molecular events.

### Immuno-metabolic dysregulation was identified during microvasculopathy to fibrosis conversion

**Figure 4:**
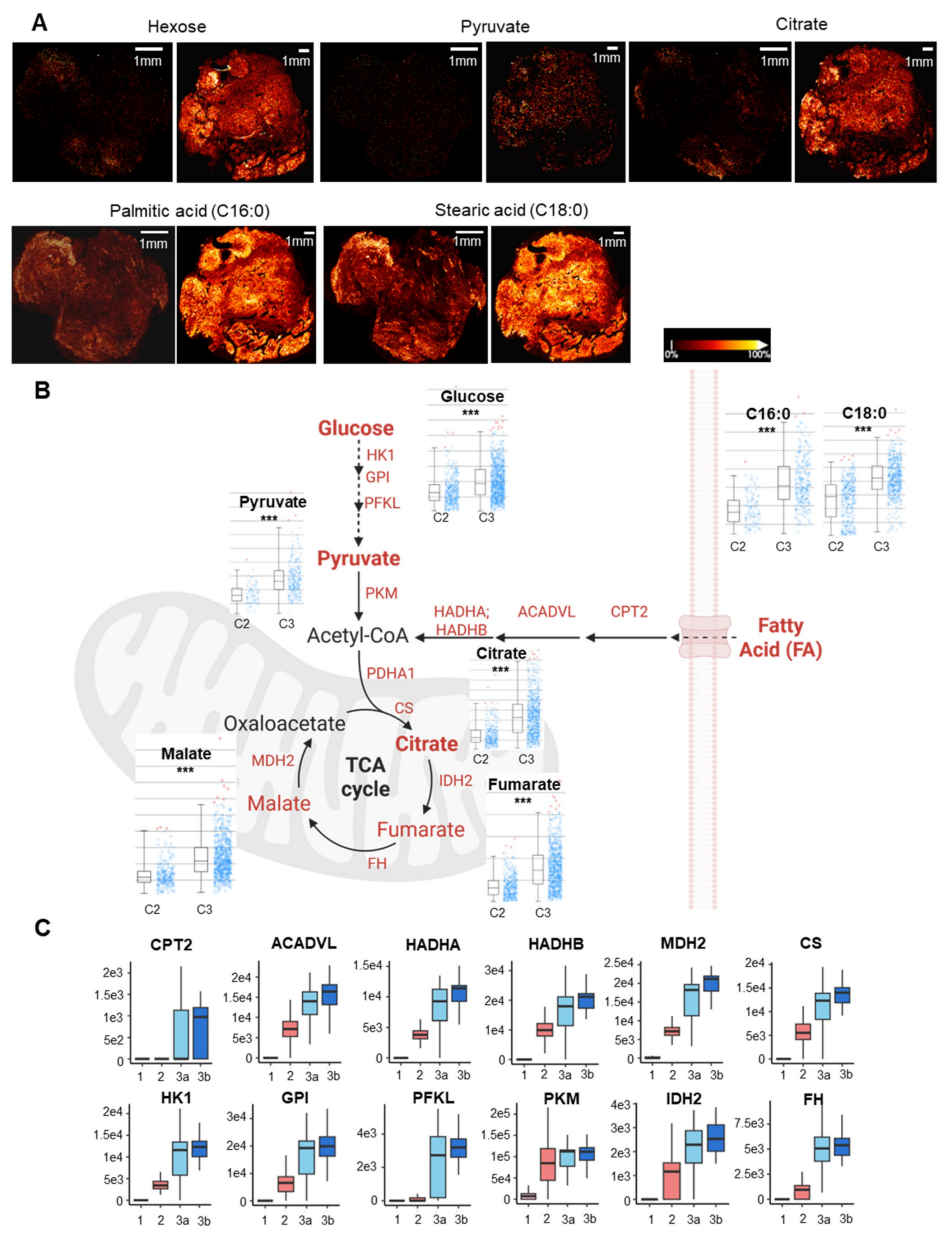
** Immunometabolic dysregulation during microvasculopathy-fibrosis conversion. A** Paired MS image of key energy metabolites of two synovium samples from adjacent section slides of the LCM-spatial proteomics sampling slides. MALDI-MSI was performed at a resolution of 20 uM. Scale bar: 1mm. Relative ion intensity is represented using a colour bar, with bright gold colour as the 100% intensity. **B** Integrated analysis of key energy metabolic pathways (beta-oxidation, glycolysis, TCA) comparing microvasculopathic and fibrotic stages. Statistical analyses for metabolites between the two stages using MSI intensity retrieved from A are shown in bar plots. Y-axis stands for relative intensities normalized to total ion count. X-axis: left C2 (microvasculopathy), right C3 (fibrosis). ***: *p* < 0.001. Red dots represent outliers. Rate-limiting enzymes up-regulated in C3 compared to C2 were marked in red. **C** Boxplots for selected metabolic regulators. The median and standard deviation of signal intensities are shown for all voxels within each functional stage. Abbreviations: HK1 Hexokinase 1; GPI Glucose-6-Phosphate Isomerase; PFKL Phosphofructokinase, Liver Type; PKM Pyruvate Kinase M1/2; HADHA/B Hydroxyacyl-CoA Dehydrogenase Trifunctional Multienzyme Complex Subunit Alpha/Beta; ACADVL Acyl-CoA Dehydrogenase Very Long Chain; CPT2 Carnitine Palmitoyltransferase 2; PDHA1 Pyruvate Dehydrogenase E1 Subunit Alpha 1; CS Citrate Synthase; IDH2 Isocitrate Dehydrogenase 2; FH Fumarate Hydratase; MDH2 Malate Dehydrogenase 2.

To explore the metabolic status between different functional niches, metabolomic imaging was performed by MALDI-MSI on consecutive slides of sample sections at a resolution of 20 *μ*m in parallel. A total of 2618 molecular features were identified, among which a comprehensive coverage of 79 energy metabolites was achieved (**Table S1**). MSI images of key energy metabolites, including hexose, pyruvate, TCA intermediates, and fatty acids, were acquired for the entire cryosection slide, broadly incorporating and encompassing the spatial proteomics sampling regions and adjacent tissues (**Figure 4A**, **Figure S6**). Significant metabolic alterations in glycolysis and beta-oxidation were observed across and within two specimens.

We focused on comparing the perturbed energy metabolism pathways between the microvasculopathic and fibrotic stages, given the identified pseudo-time trajectory in Figure 3A. Based on the quantitative analysis of metabolites' intensities from MSI in regions of c3 and c2. A significant increase in energy metabolism in cluster 3 compared to cluster 2, particularly on fatty acid regulation, was revealed (**Figure 4B**). MALDI-TOF MS/MS analysis of the metabolite standard was carried out using timsTOF flex to validate the key identified energy metabolites (**Figure S7**). Citrate, malate, and PA were confirmed based on their unique MS2 fragment ions. The observation was further echoed by the abundance of key metabolic enzymes (**Figure 4C**) and GSEA enrichment of relevant biological pathways using quantitative proteomics data (**Figure S8**). The significant dysregulation in fatty acid metabolism and inflammation revealed the involvement of metabolic inflammation during the microvasculopathy-fibrosis conversion and agreed with metabolic dysregulation as a known OA risk factor^4^, ^25^-^27^.

### Co-expression analysis across functional stages revealed potential physio-pathological events and interplays in OA

**Figure 5:**
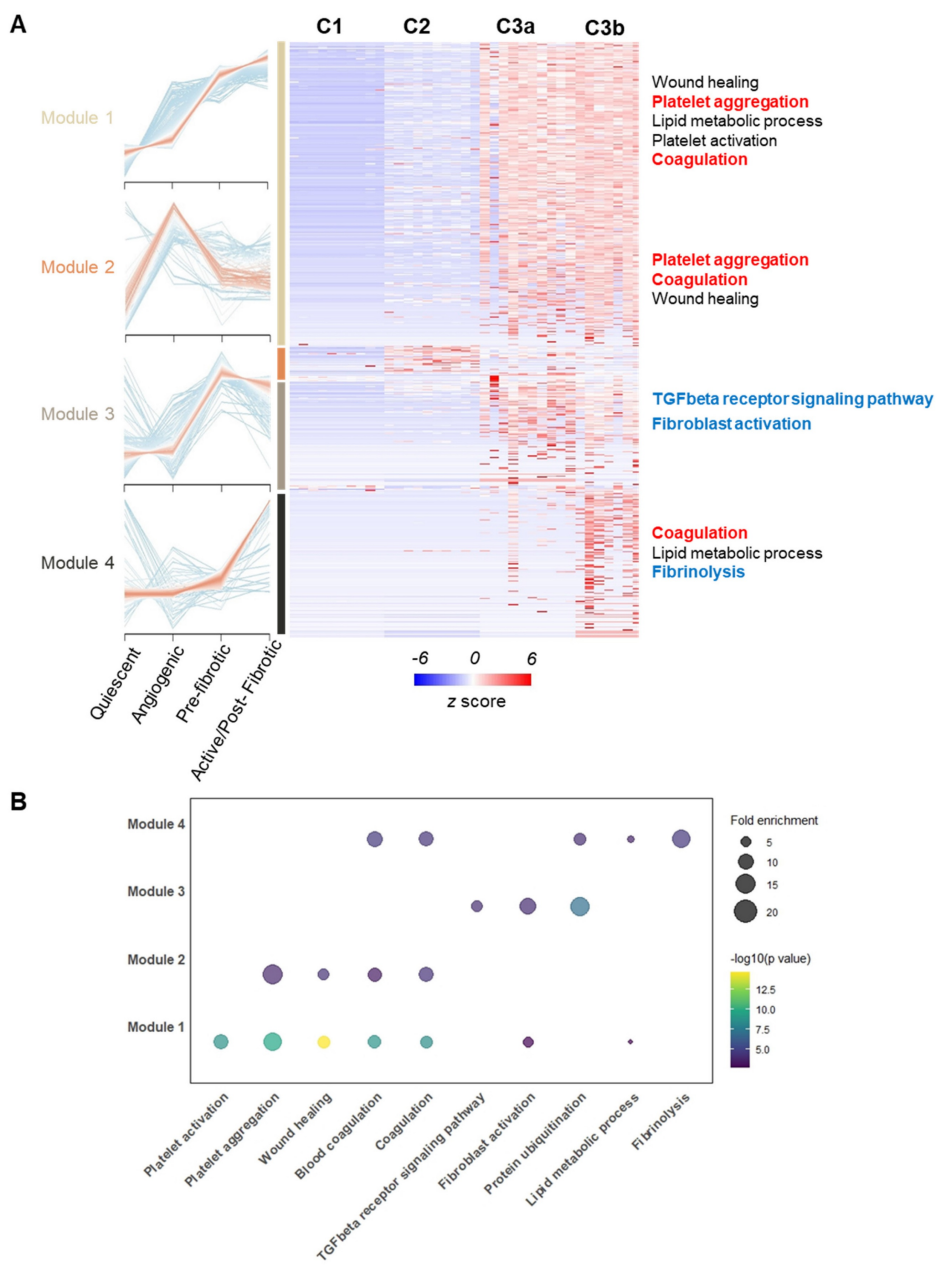
** Co-expression analysis across functional stages revealed potential physio-pathological events and interplay in OA progression. A** Four selected co-expression modules across functional stages. The heatmap displays z-score normalized protein expression levels in representative voxels (n = 8 per stage). Selected OA hallmark functional terms from Gene Ontology Biological Process (GOBP) enrichment analysis are shown. **B** Bubble plot showing GOBP enrichment analysis results for the four identified co-expression modules. Enrichment p-values were corrected using the Benjamini-Hochberg method (q < 0.01).

To investigate underlying molecular events during the observed pseudo-time trajectory conversion, we grouped all the identified proteins into modules based on Z-score normalized expression patterns across different functional stages using the mfuzz algorithm^28^. Four co-expression modules stood out due to their stage-associated patterns, which included 2060 proteins, as shown in the heatmap (**Figure 5A**). We then performed functional enrichment analysis within co-expression modules by Gene Ontology Biological Process (GOBP) (**Figure 5B**). Critically, the TGF-β pathway and fibroblast activation were enriched significantly in module 3, of which module proteins topped their expression in pre-fibrotic niches. The TGF-β pathway was a well-known driving factor for myofibroblast activation in rheumatoid tissues^29^, ^30^, which further supported the existence of a transition stage as we proposed. Intriguingly, microvascular thrombosis hallmarks, such as platelet aggregation and coagulation, were significantly enriched in modules associated with microvasculopathy and fibrosis (**Figure 5**). Our observation underscored a link between microvascular pathology (such as microthrombosis) and fibrosis, echoing the recent revisit to the vascular aetiology with OA^16^.

### Correlation analysis revealed crosstalk among platelet activity, inflammatory, and fibrotic markers

**Figure 6:**
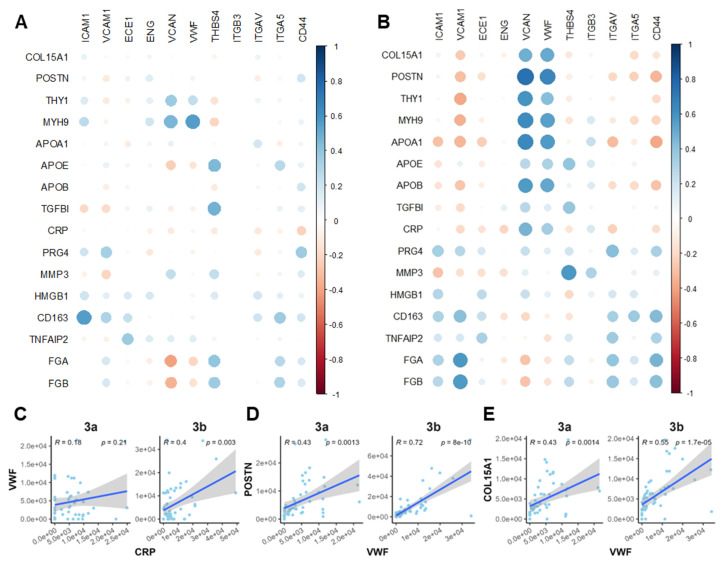
** Correlation analysis revealed crosstalk among platelet activity, inflammatory, and fibrotic markers. A-B** Correlation bubble maps of selected marker proteins in the Microvasculopathic (A) and Fibrotic (B) stages, respectively. Bubble size represents the magnitude of the correlation, and colour indicates the direction (positive or negative). **C-E** Scatter plots of representative marker pairs demonstrate fibrotic, microvasculopathic, or inflammatory relationships. Linear regression models with 95% confidence intervals are shown, along with R and *p*-values.

To further clarify the interplay among the observed OA-related events, we performed Pearson correlation analysis on a panel of markers spanning microvasculopathy, fibrosis, and platelet activity. Two functional stages (microvasculopathic and fibrotic) were chosen, and strikingly different stage-specific correlations were observed. Only scattered correlations between marker pairs were identified during the microvasculopathic stage, such as blood vessel marker vWF and fibroblast marker MYH9, or platelet marker THBS4 and fibrotic regulator TGFBI (**Figure 6A**). In contrast, significant correlation groups could be identified among blood vessel markers (vWF, VCAN) and myofibroblasts (POSTN, THY1, etc.) or inflammatory markers (APOA1, CRP, etc.) during the fibrotic stage (**Figure 6B**). By further studying the correlation patterns in pre-fibrotic stage and active/post-fibrotic stages, a clear trajectory of correlation between the representative platelet-activating, inflammatory, and fibrotic markers was demonstrated in **Figure 6C-E**. These results confirmed our discoveries in stage-specific functional niches, indicating their utility in accurate molecular endotyping for OA.

## Discussion

In this study, we present the first integrative spatial multi-omics imaging of OA-associated synovium, generated through integrated profiling of 3,534 proteins and 79 energy metabolites. Clustering of these molecular signatures identified four distinct functional endotypes, representing progressive stages of synovial pathobiology. Pseudotime trajectory analysis revealed a dynamic continuum of synovitis, beginning with endothelial dysfunction and microvascular thrombosis and advancing toward stromal expansion and myofibroblast activation. Our findings highlight the synergistic value of spatially resolved proteomics and matrix-assisted laser desorption/ionization mass spectrometry imaging (MALDI-MSI) for high-dimensional tissue phenotyping. This integrative approach establishes a molecular framework for synovial endotyping in OA and lays the groundwork for the development of personalized, endotype-targeted interventions.

Biochemical alterations often precede the onset of overt morphological changes^18^, ^31^, highlighting the need for multi-layered data to achieve accurate molecular classification^32^. By integrating morphological and molecular information, we delineated a transitional, pre-fibrotic stage that would otherwise be mislabelled as purely microvasculopathic or fibrotic through single-modality analyses. This more nuanced classification captures key transitional states in the microvasculopathic-to-fibrotic continuum driven by TGF-β-mediated fibroblast activation, in alignment with previous mechanistic evidence^33^, ^34^.

Our findings highlight immunometabolic dysregulation, particularly in lipid metabolism, as a key amplifier of the microvasculopathy-to-fibrosis transition, reinforcing the idea that OA is a metabolic syndrome^16^, ^26^, ^35^. Consistent with the microvascular aetiology hypothesis^36^, we also implicate coagulation-driven microvascular dysfunction as a critical upstream event. Hypoxia-induced activation of NF-κB and nitric oxide signalling pathways appear central in linking microthrombosis and altered energy metabolism^16^, ^35^, opening potential avenues for developing therapeutic strategies aimed at halting progression toward irreversible fibrosis^37^, ^38^.

Technological enhancements are pivotal for gaining a deeper understanding of the molecular and cellular events that occur in OA, but methodological optimizations are critical for achieving these insights. While common workflows often focus on lipid imaging in OA^39^, ^40^, our optimised MALDI-MSI platform enabled coverage of energy metabolites in the low m/z range, utilizing the satisfactory performance of N-(1-naphthyl)ethylenediamine dihydrochloride (NEDC) as matrix for energy metabolites and small polar compounds^41^. Additionally, we implemented a micro-flow LC strategy on a short UHPLC column to reduce running times to one-third of previous protocols^13^, ^15^, substantially enhancing throughput in spatial proteomics. This approach, previously associated with increased robustness and analytical efficiency in proteomic workflows^42^, ^43^, imposed a higher sample demand, which we offset by maintaining a 200 µm spatial resolution. Consequently, we analyzed 15mm^2^ tissue areas within 4 days of LC/MS running time, aligning with the throughput of MALDI-MSI and histopathological processing, and readily translatable to arthroscopic biopsy workflows. Preliminary data further suggest the feasibility of higher spatial resolution down to 50 µm and shorter LC gradients of 7 minutes, enabling over 160 samples per day, offering promising avenues for future optimization.

Taken together, our findings reveal the coordinated evolution of proteomic and metabolic landscapes across distinct synovial pathologies, establishing a robust foundation for a precision medicine paradigm that integrates spatial multi-omics with clinical stratification for enhanced clinical trials and better patient care. This integrative framework not only enables tailored therapeutic targeting but also offers a path to address comorbidities and OA disease heterogeneity systematically. With the potential to reshape current paradigms in OA diagnosis and treatment, this approach represents a decisive step toward individualized care and disease management. Clearly, due to the proof-of-concept nature of the study, validation in larger, stratified patient cohorts will be critical to refine these findings and accelerate their translation into clinical practice.

## Methods

### Ethics and biosafety

The investigation complied with the principles outlined in the Declaration of Helsinki. Human osteoarthritic knee synovium samples were obtained as surgical waste from the Queen Mary Hospital. Informed consent was obtained before the surgery. Clinic ethics approval has been approved by the Institutional Review Board of the University of Hong Kong/Hospital Authority Hong Kong West Cluster (HKU/HA HKW IRB, Ref. No. UW 23-596). Non-clinic human research ethics, biological safety, and chemical safety approval were approved by the Research Ethics Committee of Hong Kong Baptist University (REC/24-25/0031).

### Tissue preparation and histological staining

Sex and gender were not considered in the study design due to the case study nature. Two representative regions (Figure 2B and 2A, respectively), from a single OA patient of the lateral proximal side were isolated upon total knee replacement surgery and immediately placed in ice-cold saline. The surgeon eye-judged the level of inflammation based on the degree of tissue vasculature (white or pink/red), as we previously described^44^.

Excess adipose tissue was dissected from the synovium. The tissue was embedded in 5% carboxymethylcellulose (Sigma-Aldrich), snap-frozen in liquid nitrogen, and stored at -80°C until cryosectioning. 10 μm-thick sections were thaw-mounted onto polyethylene naphthalate (PEN) membrane slides (Leica). Consecutive sections were stained with haematoxylin, aniline blue, and ponceau S using a Modified Masson Trichrome Stain kit (Solarbio) according to the manufacturer's instructions. Stained sections were imaged at 20X magnification using a Zeiss Axioscan 3 slide scanner.

### Laser capture microdissection (LCM)

Regions of interest (ROIs) for microdissection were selected based on Masson's trichrome-stained images. A grid of 300 pre-defined 200 μm x 200 μm squares was generated using Aivia AI (Leica) and registered to the PEN membrane slide. Using a 20X objective (HC PL FL L 20x/0.40 CORR) in middle pulse mode, individual voxels were laser micro-dissected. The following laser settings were used: power 45, aperture 1, speed 11, middle pulse count 2, final pulse 1, head current 68%, pulse frequency 637, and offset 110. To minimize electrostatic interference, slides were rinsed three times with pure ethanol before dissection. Dissected voxels were collected into 2 μL droplets of dimethyl sulfoxide (DMSO) pre-loaded in the caps of 8-well PCR strips. Each voxel's spatial location (row and column) was recorded for subsequent image reconstruction. Collected samples were freeze-dried in a lyophilizer and stored at -80°C until proteolytic digestion.

### Spatial proteomic sample preparation and LC-MS/MS analysis

Tryptic digestion for LCM dissected samples follows our published method with minor adaptations.^22^, ^45^ Briefly, 20 μL of 100 mM triethylammonium bicarbonate (TEABC, Sigma-Aldrich) was added to each sample-containing PCR tube. Samples were vortexed, centrifuged at 4500 x g for 10 minutes, and heated to 95°C to solubilize proteins. As combination of trypsin and Lys-C proteases is well-established in proteomics to improve digestion efficiency and reduce missed cleavages,^46^ 1 μL of 20 ng/μL trypsin/Lys-C mix (Promega) in 100 mM TEABC was added, followed by vigorous vortexing and centrifugation at 4500 x g for 10 minutes. Proteolytic digestion was performed at 37°C for 12 hours. Digestion was quenched by adding formic acid (MS grade, Thermo Fisher Scientific) to a final concentration of 1%. Peptides were dried using a SpeedVac concentrator and stored at -80°C until LC-MS/MS analysis.

Dried peptides were reconstituted in 3 μL of 2% acetonitrile/0.1% formic acid and loaded onto a 5 cm (length) x 75 μm (i.d.) column packed with 1.7 μm C18 modified silica beads (Ionopticks, Australia). Peptides were separated using a 15-minute linear gradient of 2-25% solvent B (0.1% formic acid in acetonitrile) over 12 minutes, followed by a 1-minute ramp to 95% solvent B, which was held for 2 minutes. A constant flow rate of 1 μL/min was maintained, and the column temperature was set to 50°C. Mass spectrometry analysis was performed on a timsTOF flex MALDI2 (Bruker Daltonics) instrument operated in DIA-PASEF mode. Data acquisition was done using default DIA-PASEF parameters except the following: 25 m/z isolation window (400-1200 Da), 3 ion mobility windows (1/K_0_ 0.6-1.3), 50 ms ramp time, and 16 PASEF frames per full MS scan, resulting in a cycle time of less than 1 second.

### Proteomic data processing and database search

Spatial proteomics data files of each voxel, named according to numeric spatial coordinates, were annotated with histological information derived from Masson's trichrome staining, including total collagen (blueness), inflammation (redness) within each voxel using ImageJ software.

Database searches for spatial-resolved proteomics samples were then performed using DIA-NN in spectral library mode with the match-between-runs option enabled^47^. Cysteine carbamidomethylation was removed from fixed modification, with all other settings left as default. To construct the spectral library, a pooled synovium peptide sample was fractionated using high pH reversed-phase fractionation (Thermo Fisher Scientific) and analysed using the same LC condition (15 min gradient on a 5 cm long column) on the same mass spectrometer operated in data-dependent acquisition (DDA) mode. The DDA file was searched using MS Fragger^48^ with default settings against Uniprot human database (Homo sapiens, 2024) except no cysteine carbamidomethylation allowed.

### Protein spatial imaging

Protein expression levels were log2 transformed and visualized concerning their spatial index (numeric spatial coordinates recorded in spatial proteomics data) using the pheatmap package in R.

### Data preprocessing and clustering

A complete input matrix is required for UMAP dimension reduction and visualization. Blank voxels were excluded and marked as cluster 0, leaving 390 voxel data from two slides for further analysis. Proteins identified in less than 40% (156 out of 390) voxels across the experiment were removed from further analysis. The remaining missing values of signal intensity were arbitrarily imputed as 1. UMAP dimensionality reduction was performed on the imputed data using the uwot package in R/Bioconductor, number of neighbours (n_neighbours) was set as 20. Clustering was then performed using either the K-means algorithm with 4 centres. The Voxel dataset was then visualized on a 2D-UMAP using ggplot2 (v. 3.5.1).

### Co-expression and pathway analysis

Fuzzy c-means algorithm (mfuzz v2.6.1)^28^ was applied to identify proteins with similar expression trends across different functional stages with varying degrees of association. The fold change (FC) of each protein compared to its signal intensity in cluster 1 (quiescent cluster) was used as the input matrix. A default value with 6 centres (modules) was used.

A total of 2060 proteins belonging to four mfuzz modules were then used to construct the expression heatmap. Expression patterns in representative voxels in each functional cluster (n=8) were visualized by co-expression clustering using the pheatmap package in R. Expression level was shown in z-score converted from the signal intensity.

Pathway analysis for proteins belonging to each module was then conducted by clusterProfiler (v.4.12.6) using the Gene Ontology Biological Process (GOBP) database (Genome-wide annotation for human from org.hs.eg.db, v.3.19.1). The *p*-values were corrected using the Benjamini-Hochberg method with a q cut-off value of 0.01. The entire set of proteins detected in the experiment was used as the background set. The selected enriched terms were visualized in a 2D-bubble plot using the enrichplot package (v.1.24.4).

### Correlation analysis

The protein expression matrix in each voxel was used for Spearman's correlation analysis. The correlation score between protein markers was calculated by the rcorr package based on the signal intensity from the dataset. A correlation bubble map was then generated to visualize the relationships among selected marker proteins using corrplot (v.0.95). For the scattered plots representing one-to-one correlation between selected marker sets, the R was calculated by fitting linear models with a 95% confidence interval and plotted by ggplot.

### MALDI-mass spectrometry imaging (MALDI-MSI)

Collected synovial tissue was snap-frozen in liquid nitrogen and stored at -80°C. Sections of 10 μm thickness were thaw-mounted onto indium tin oxide-coated glass slides and dried under vacuum for at least 15 minutes. Slides were pre-scanned using an optical scanner for image registration. A matrix solution of 5 mg/mL N-(1-naphthyl)ethylenediamine dihydrochloride (NEDC) in methanol was sonicated for 10 minutes and applied to the slides in ten layers using an in-house electrospray device.^49^ The following key instrumental parameters were applied: flow rate of 0.05 mL/min; tracking spacing of 2 mm; 25 s drying time; temperature of the spray head 66 °C.

Mass spectrometry imaging was conducted on a timsTOF flex MALDI 2 instrument (Bruker Daltonics) as reported. A spatial resolution of 20 *μ*m was achieved. Mass spectra were acquired between *m*/*z* 80 and 2000 in negative ion reflector mode. Key parameters were optimized and applied throughout the experiment, including averaging of 1000 shots, a 20.84 kV reflector voltage, 20 kV source voltage, and a pulsed ion extraction time of 100 ns. Calibration was performed using external standards (Bruker Daltonics) as reported. At least three spectra per representative region were averaged using DataAnalysis software. Peaks were identified by searching against the Human Metabolome Database with a mass tolerance of 10 ppm. Imaging data and metabolite assignments were imported into SCiLSlab software for visualization and segmentation using the bisecting K-means algorithm.

Metabolite quantification was performed using SCiLS Lab software following our previous reports.^22^, ^49^ The metabolite heatmaps were firstly normalized based on total ion count. Regions of interest (ROIs) corresponding to synovial microenvironments characterized as fibrosis or angiogenesis were delineated directly on SCiLS Lab by overlaying histological and molecular images. For each ROI, the intensity values of individual pixels (voxels) were extracted and treated as independent data points for statistical analysis. Intensity distributions were visualized using box plots, and statistical significance was assessed with p-values adjusted for multiple testing using the Benjamini-Hochberg procedure, applying a false discovery rate (q) cutoff of 0.01.

## Supplementary Material

Supplementary figures and tables.
