# Supplementary material for: Integrative spatially resolved proteomic and metabolomic imaging reveals synovitis endotypes implicated in osteoarthritis progression
Source: Theranostics. 2025 Aug 30;15(18):9729–41. doi: 10.7150/thno.117788 (PMC12486396; doi:10.7150/thno.117788)
Supplement: Supplementary file 1 — Supplementary figures and tables. [file thnov15p9729s1.pdf]

Supplementary Information for
Integrative Spatially Resolved Proteomic and Metabolomic Imaging Reveals Synovitis Endotypes Implicated in Osteoarthritis Progression

Lin Zhu,[1#,*] Xin Diao,[1#] Chenrui Yuan,[1#] Chun Man Lawrence Lau,[2] Jianing Wang, [1] Wenlong Wu, [1] Ali Mobasheri,[3,4,5,6] Xavier Houard,[7] Chunyi Wen,[8*] Zongwei Cai[1,9*]

1 Department of Chemistry, Hong Kong Baptist University, Hong Kong SAR, 999077, China
2. Department of Orthopaedics and Traumatology, Li Ka Shing School of Medicine, The University of Hong Kong, Pokfulam, HKSAR, China
3. Research Unit of Medical Imaging, Physics and Technology, Faculty of Medicine, University of Oulu, PO Box 5000, FI-90014 Oulu, Finland
4. Department of Regenerative Medicine, State Research Institute Centre for Innovative Medicine, Vilnius, Lithuania
5. Department of Joint Surgery, First Affiliated Hospital of Sun Yat-sen University, Guangzhou, Guangdong, China
6. Faculty of Medicine, Université de Liège, Liège, Belgium
7. Sorbonne Université, INSERM, Centre de Recherche Saint-Antoine, CRSA, F-75012, Paris, France
8. Department of Biomedical Engineering, The Hong Kong Polytechnic University, Hung Hom, Hong Kong, China
9. Eastern Institute of Technology, Ningbo, 315200, China

# These authors contributed equally: Lin Zhu, Xin Diao, Chenrui Yuan.
* Corresponding authors: Lin Zhu (lommelfox@gmail.com; zhu_lin@hkbu.edu.hk) 0000-0002-2801-3626, Chunyi Wen (chunyi.wen@polyu.edu.hk) 0000-0003-1949-7822, Zongwei Cai (zwcai@hkbu.edu.hk) 0000-0002-8724-7684.

Supplementary Table 1. List of assigned metabolites in MSI

Assignment	Experiment m/z	Theoretical m/z	Relative error (ppm)	Adduction	Description
Taurine	124.0076	124.0077	-0.89	[M-H]-	Amino acid and Derivative
Alanine	88.0401	88.0404	-3.07	[M-H]-	Amino acid and Derivative
Creatine	130.0615	130.0619	-2.77	[M-H]-	Amino acid and Derivative
Glutamate	146.0460	146.0453	4.52	[M-H]-	Amino acid and Derivative
Glutathione	306.0757	306.0765	-2.65	[M-H]-	Amino acid and Derivative
Histidine	154.0618	154.0625	-4.22	[M-H]-	Amino acid and Derivative
Histidylalanine	225.0995	225.0995	0.04	[M-H]-	Amino acid and Derivative
Ornithine	131.0826	131.0825	1.14	[M-H]-	Amino acid and Derivative
Threonine	100.0403	100.0401	2.00	[M-H20-H]-	Amino acid and Derivative
Tryptophan	203.0836	203.0834	0.89	[M-H]-	Amino acid and Derivative
γ-Aminobutyric acid	102.0561	102.0558	3.14	[M-H]-	Amino acid and Derivative
3-Phosphoglyceric acid	184.9846	184.9851	-2.70	[M-H]-	Carbohydrates and Glycolysis
Fructose 1,6-bisphosphate	338.9890	338.9885	1.39	[M-H]-	Carbohydrates and Glycolysis
Fructose-6-Phosphate	259.0219	259.0224	-1.74	[M-H]-	Carbohydrates and Glycolysis
Glucose	215.0322	215.0328	-2.74	[M+Cl]-	Carbohydrates and Glycolysis
Glycerol 3-phosphate	171.0058	171.0065	-4.15	[M-H]-	Carbohydrates and Glycolysis
Pyruvic acid	122.9850	122.9856	-4.55	[M+Cl]-	Carbohydrates and Glycolysis
Ribose-5-phosphate	211.0010	211.0007	1.42	[M-H20-H]-	Carbohydrates and Glycolysis
Calcium	144.8694	144.8692	1.38	[M+Cl]-	Inorganic
H2SO4	96.9601	96.9601	-0.03	[M-H]-	Inorganic
H3PO4	96.9692	96.9696	-4.32	[M-H]-	Inorganic
Iron (III)	197.8082	197.8079	1.52	[M+Cl]-	Inorganic
Iron(II)	160.8420	160.8418	1.24	[M+Cl]-	Inorganic
Magnesium	130.8894	130.8892	1.53	[M+Cl]-	Inorganic
Potassium	108.9014	108.9018	-3.67	[M+Cl]-	Inorganic
Sodium	92.9273	92.9277	-4.30	[M+Cl]-	Inorganic
Zinc	170.8324	170.8330	-3.51	[M+Cl]-	Inorganic
FA 16:0	255.2332	255.2335	-0.98	[M-H]-	Lipids and Fatty acid
FA 18:0	283.2649	283.2635	4.94	[M-H]-	Lipids and Fatty acid
FA 18:1	281.2482	281.2481	0.36	[M-H]-	Lipids and Fatty acid
FA 18:2	279.2319	279.2329	-3.58	[M-H]-	Lipids and Fatty acid
FA 20:1	309.2802	309.2791	3.56	[M-H]-	Lipids and Fatty acid
FA 20:2	307.2656	307.2634	7.16	[M-H]-	Lipids and Fatty acid
FA 20:2;O2	375.2312	375.2305	1.87	[M+Cl]-	Lipids and Fatty acid
FA 20:4	303.2333	303.2328	1.65	[M-H]-	Lipids and Fatty acid
FA 20:4;O	319.2286	319.2273	4.07	[M-H]-	Lipids and Fatty acid
FA 20:5	301.2175	301.2169	1.99	[M-H]-	Lipids and Fatty acid
FA 22:4	331.2646	331.2639	2.11	[M-H]-	Lipids and Fatty acid
FA 22:5	329.2484	329.2485	-0.30	[M-H]-	Lipids and Fatty acid
FA 22:6	327.2330	327.2326	1.22	[M-H]-	Lipids and Fatty acid
FA 22:7	361.1912	361.1929	-4.57	[M+Cl]-	Lipids and Fatty acid
Glycerophosphoinositol	315.0488	315.0479	2.86	[M-H20-H]-	Lipids and Fatty acid
LPA 16:0	409.2382	409.2362	4.86	[M-H]-	Lipids and Fatty acid

LPA 18:0	437.2680	437.2660	4.69	[M-H]-	Lipids and Fatty acid
LPA 18:1	435.2508	435.2498	2.21	[M-H]-	Lipids and Fatty acid
LPA 18:2	433.2359	433.2352	1.62	[M-H]-	Lipids and Fatty acid
LPA 20:4	457.2350	457.2353	-0.66	[M-H]-	Lipids and Fatty acid
LPE 16:0	452.2765	452.2783	-3.98	[M-H]-	Lipids and Fatty acid
LPE 18:0	480.3079	480.3080	-0.21	[M-H]-	Lipids and Fatty acid
LPE 18:1	478.2973	478.2953	4.18	[M-H]-	Lipids and Fatty acid
LPE 18:2	476.2807	476.2797	2.10	[M-H]-	Lipids and Fatty acid
LPE 20:0	508.3410	508.3400	1.97	[M-H]-	Lipids and Fatty acid
LPE 20:4	500.2772	500.2783	-2.20	[M-H]-	Lipids and Fatty acid
LPE O-16:1	436.2836	436.2817	4.35	[M-H]-	Lipids and Fatty acid
LPE O-18:1	464.3170	464.3149	4.52	[M-H]-	Lipids and Fatty acid
LPG 16:0	483.2734	483.2736	-0.41	[M-H]-	Lipids and Fatty acid
LPI 18:0	599.3210	599.3186	4.00	[M-H]-	Lipids and Fatty acid
LPI O-16:0	557.3076	557.3081	-0.90	[M-H]-	Lipids and Fatty acid
LPS 20:3	546.2831	546.2822	1.65	[M-H]-	Lipids and Fatty acid
LPS O-16:1	480.2743	480.2727	3.29	[M-H]-	Lipids and Fatty acid
Phosphodimethylethan olamine	168.0435	168.0432	2.08	[M-H]-	Lipids and Fatty acid
Adenosine diphosphate	426.0226	426.0221	1.08	[M-H]-	Nucleotides and Derivative
Adenosine monophosphate	346.0567	346.0567	-0.03	[M-H]-	Nucleotides and Derivative
Dihydrothymine	109.0407	109.0402	4.59	[M-H20-H]-	Nucleotides and Derivative
Inosinic acid	347.0409	347.0395	3.92	[M-H]-	Nucleotides and Derivative
Uric acid	167.0215	167.0219	-2.22	[M-H]-	Nucleotides and Derivative
Uridine 5'-monophosphate	323.0289	323.0275	4.40	[M-H]-	Nucleotides and Derivative
Ethyl hydrogen sulfate	106.9806	106.9805	0.93	[M-H20-H]-	Organic acid
Glutaric acid	131.0348	131.0349	-0.84	[M-H]-	Organic acid
O-Phosphoethanolamine	140.0119	140.0116	2.36	[M-H]-	Organic acid
Heme	615.1692	615.1700	-1.30	[M-H]-	Other
inositol cyclic phosphate	241.0110	241.0121	-4.73	[M-H]-	Other
Methylisocitric acid	241.0110	241.0121	-4.73	[M-H]-	Other
Phosphonoacetate	138.9797	138.9802	-3.53	[M-H]-	Other
Pyrrole-2-carboxylic acid	110.0250	110.0248	1.82	[M-H]-	Other
2-Ketobutyric acid	101.0240	101.0244	-3.76	[M-H]-	TCA Cycle
Citric acid	191.0199	191.0204	-2.72	[M-H]-	TCA Cycle
Fumaric acid	115.0041	115.0037	3.63	[M-H]-	TCA Cycle
Malic acid	133.0144	133.0142	1.15	[M-H]-	TCA Cycle

Supplementary Table 2. Protein Abbreviations and Full Names

Abbreviations	Full name	Abbreviations	Full name
FGA	fibrinogen alpha chain	HK1	Hexokinase 1
FGB	fibrinogen beta chain	IL6	Interleukin 6
COL15A1	Collagen Type XV Alpha 1 Chain	IL18	Interleukin 18
TGFBI	Transforming growth factor beta induced	ENG	Endoglin
THY1	Thy-1 Cell Surface Antigen	VIM	Vimentin
VCAM1	Vascular cell adhesion protein 1	CS	Citrate Synthase
HRG	Histidine Rich Glycoprotein	FH	Fumarate Hydratase
MMP3	Matrix Metallopeptidase 3	C3	Complement C3
HMGB1	High Mobility Group Box 1	C9	Complement C9
TGFB1I1	Transforming Growth Factor Beta 1 Induced Transcript 1	PRDX1	Peroxiredoxin 1
S100A9	S100 Calcium Binding Protein A9	IL22	Interleukin 22
HSPA1A	Heat Shock Protein Family A (Hsp70) Member 1A	IL13	Interleukin 13
SERPINA1	Serpin Family A Member 1	IL15	Interleukin 15
GPI	Glucose-6-Phosphate Isomerase	IL12A	Interleukin 12A
PFKL	Phosphofructokinase, Liver Type	IL10	Interleukin 10
PKM	Pyruvate Kinase M1/2	PROCR	Protein C Receptor
HADHA	Hydroxyacyl-CoA Dehydrogenase Trifunctional Multienzyme Complex Subunit Alpha	FGG	fibrinogen gamma chain
HADHB	Hydroxyacyl-CoA Dehydrogenase Trifunctional Multienzyme Complex Subunit Beta	CRP	C-Reactive Protein
ACADVL	Acyl-CoA Dehydrogenase Very Long Chain	ALB	Albumin
CPT2	Carnitine Palmitoyltransferase 2	PGM1	phosphoglucomutase 1
PDHA1	Pyruvate Dehydrogenase E1 Subunit Alpha 1	PRG4	Proteoglycan 4
IDH2	Isocitrate Dehydrogenase 2	THBS4	Thrombospondin 4
SLC27A1	Solute Carrier Family 27 Member 1	FN1	Fibronectin 1
SLC25A11	Solute Carrier Family 25 Member 11	VWF	Von Willebrand Factor
F2	Coagulation Factor II, Thrombin	ENO1	Enolase 1
SERPINC1	Serpin Family C Member 1	KNG1	kininogen 1
ICAM1	Intercellular Adhesion Molecule 1	MDH2	Malate Dehydrogenase 2
ECE1	Endothelin Converting Enzyme 1	APOA1	Apolipoprotein A1
IL1RN	Interleukin 1 Receptor Antagonist	APOE	Apolipoprotein E
TNFAIP2	TNF Alpha Induced Protein 2	APOB	Apolipoprotein B
MYH9	myosin, heavy chain 9	ITGB2	Integrin Subunit Beta 2
COL1A1	Collagen Type I Alpha 1 Chain	ITGB3	Integrin Subunit Beta 3
COL3A1	Collagen Type III Alpha 1 Chain	ITGAV	Integrin Subunit alpha V
PDGFRA	Platelet Derived Growth Factor Receptor Alpha	ITGA5	Integrin Subunit alpha 5

Supplementary Figure S1. Histological staining of representative synovium samples used in the study.

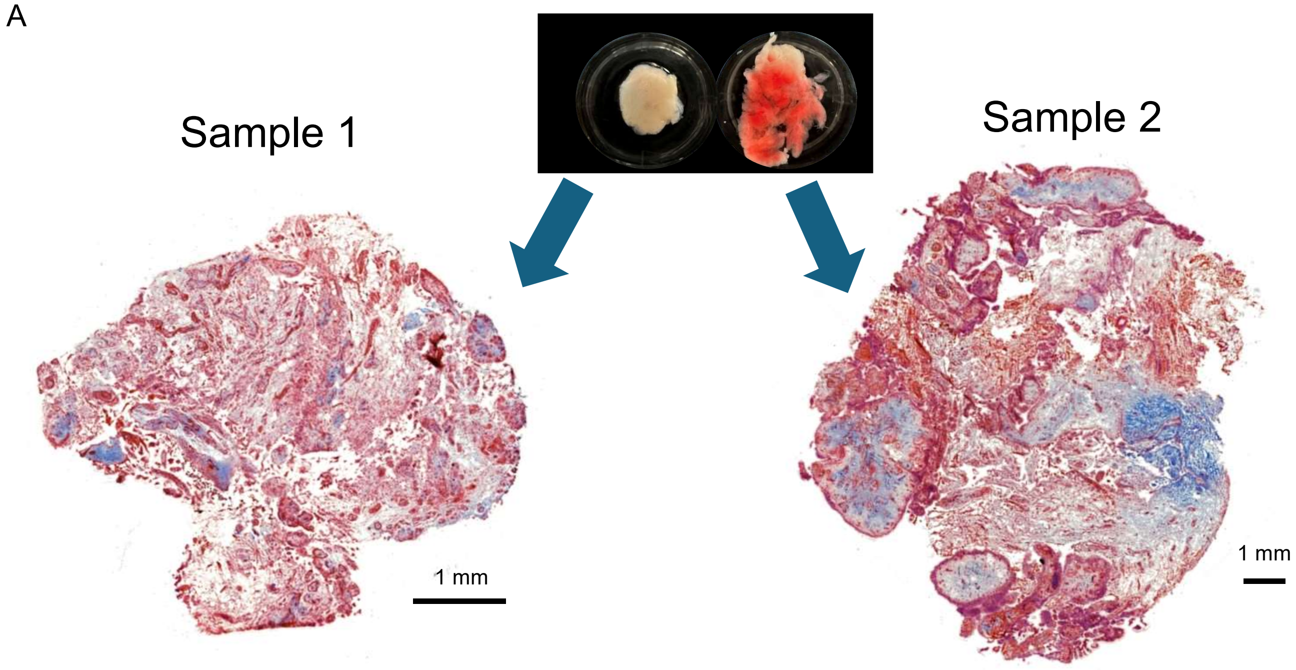

Section slides by MTS from inflamed (right) and less-inflamed (left) regions of synovium specimen from one single patient receiving total knee replacement.

Supplementary Figure S2

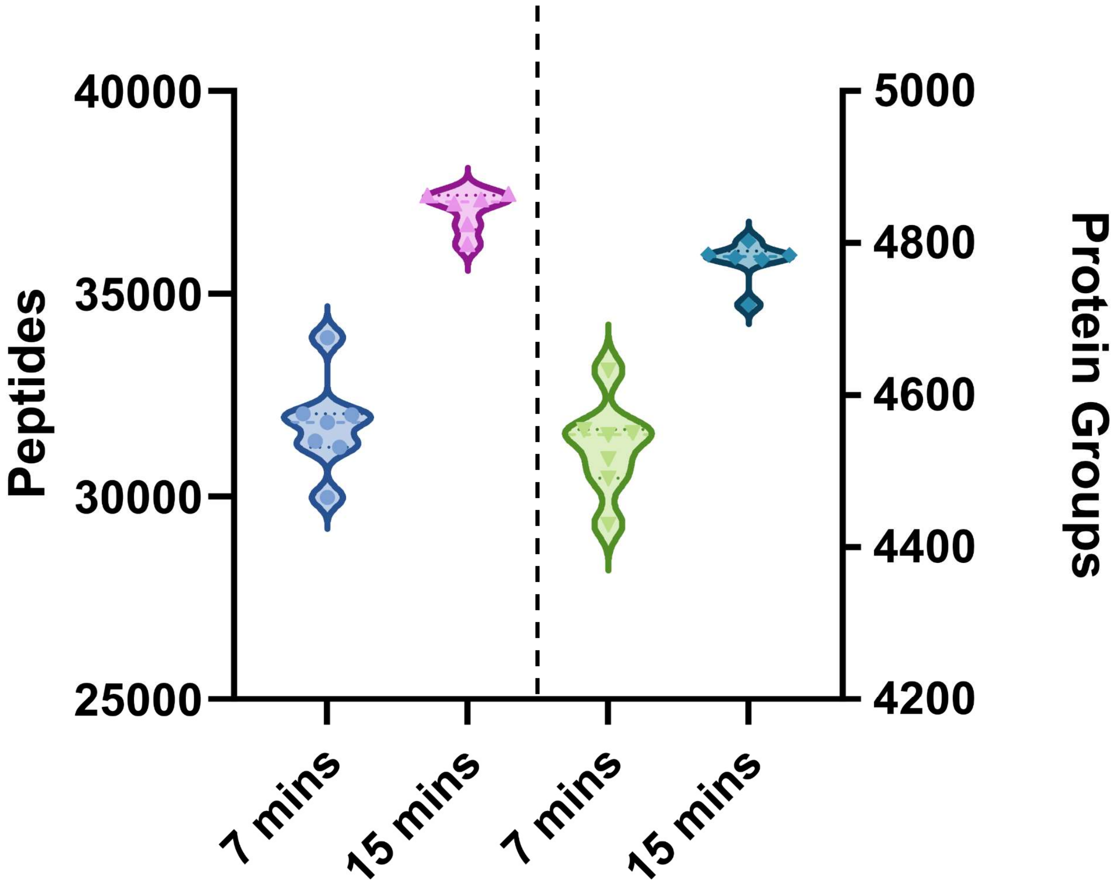

Number of identified peptides (left) and proteins (right) in 7 or 15 min gradient on a 5cm long C18 microflow UHPLC analytic column (Ionopticks, 150 um ID, 1.7 um C18).

Supplementary Figure S3

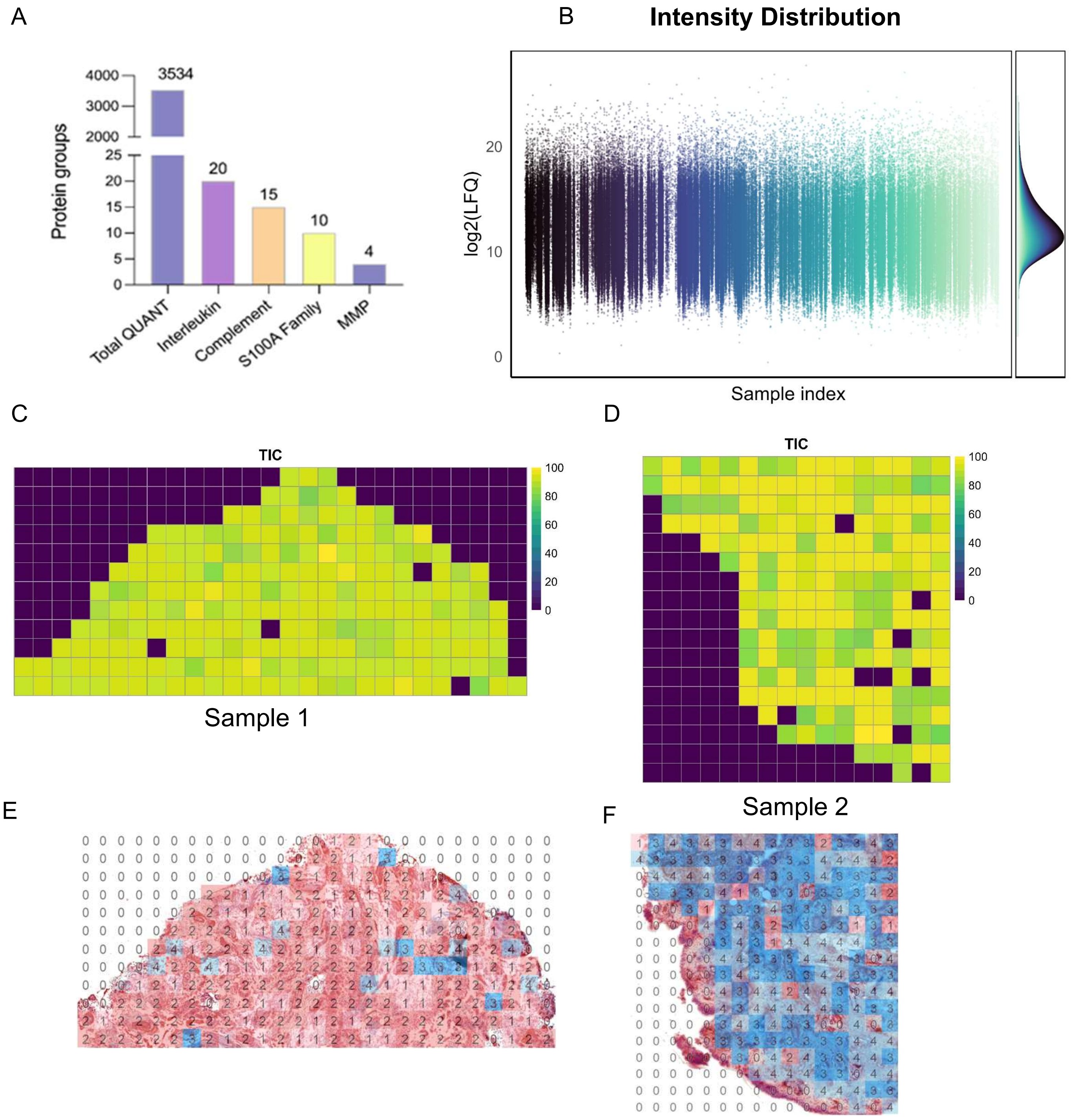

A Bar chart for all identified protein groups in the spatial resolved proteomics analysis. **B** Scatter plot for protein intensity (label-free quantitation, LFQ) in all voxel samples. **C-D** Heatmaps plotted for total intensity counts (TIC) of all identified proteins within each voxel in samples 1 and 2, based on their spatial coordinates. **E-F** Overlaid images of spatial mapping of cluster assignments with their corresponding histological staining.

Supplementary Figure S4

a. ECM remodeling, MFA, and fibrosis related markers

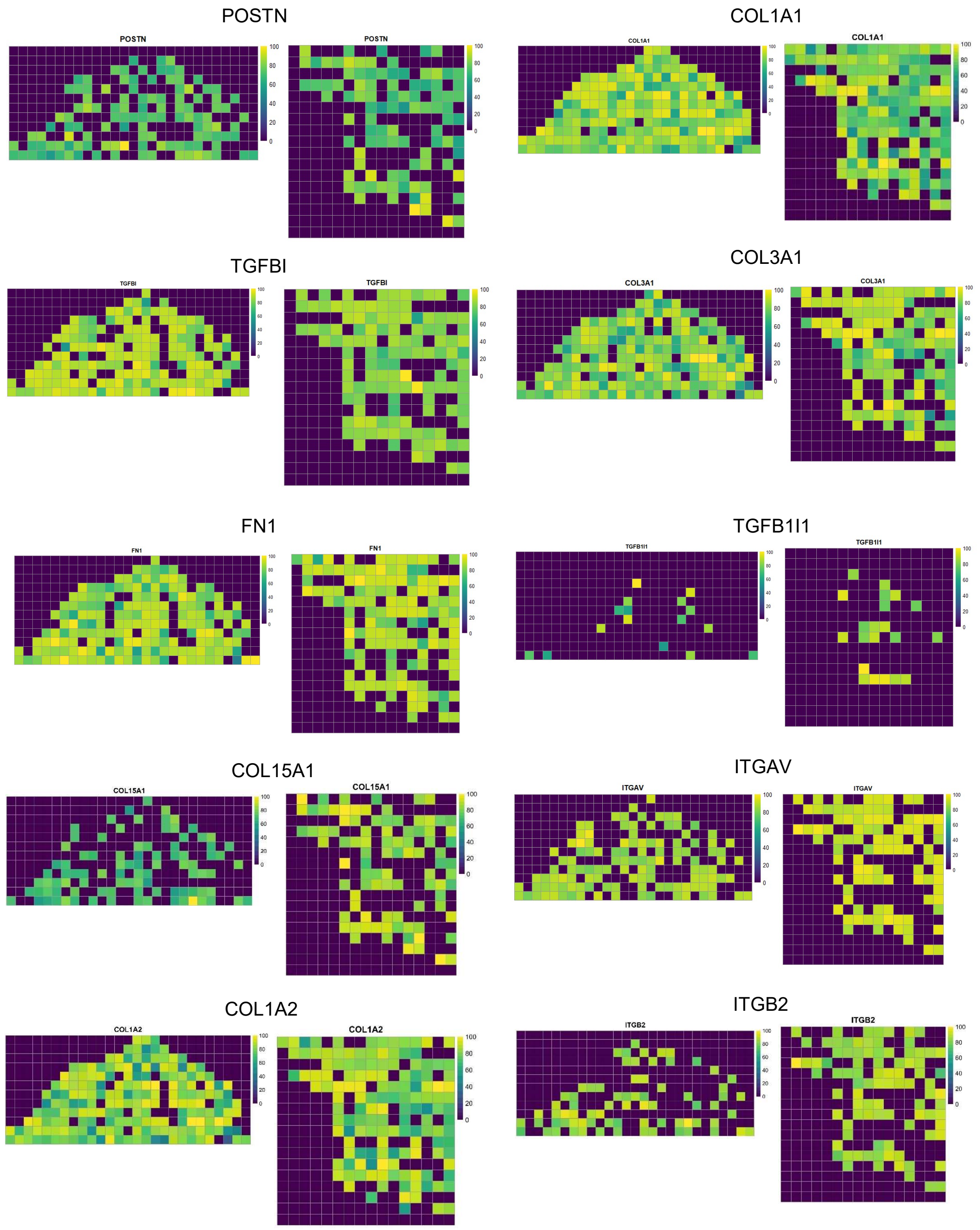

Protein expression levels within each voxel were log2 transformed and plotted according to relative intensities regarding their spatial coordinates. Gene symbol was used as abbreviations for proteins. The full name of the proteins can be found in the supplementary table S2.

S4a. (continued) ECM remodeling, MFA, and fibrosis related markers

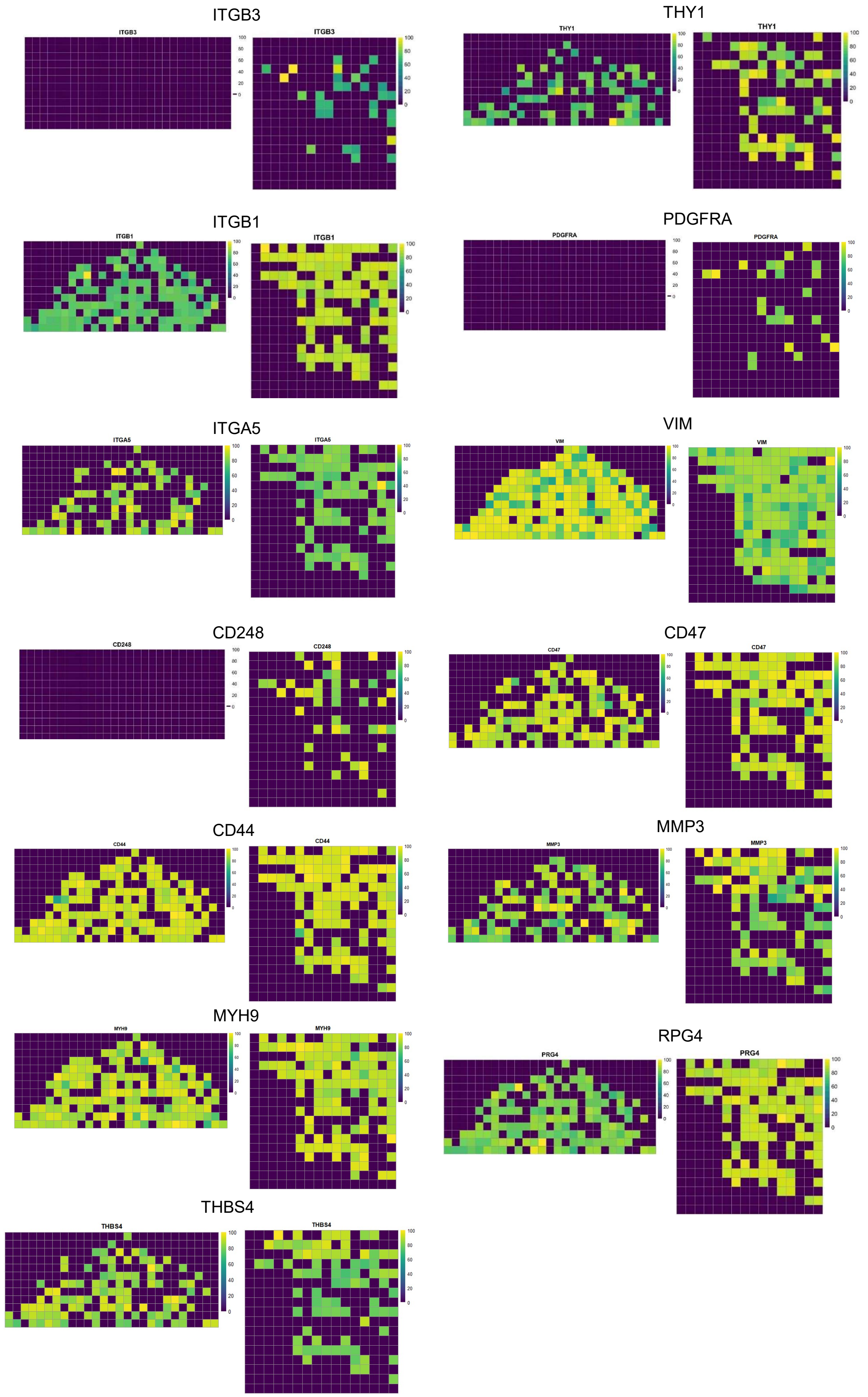

S4b. Inflammation and angiogenesis markers

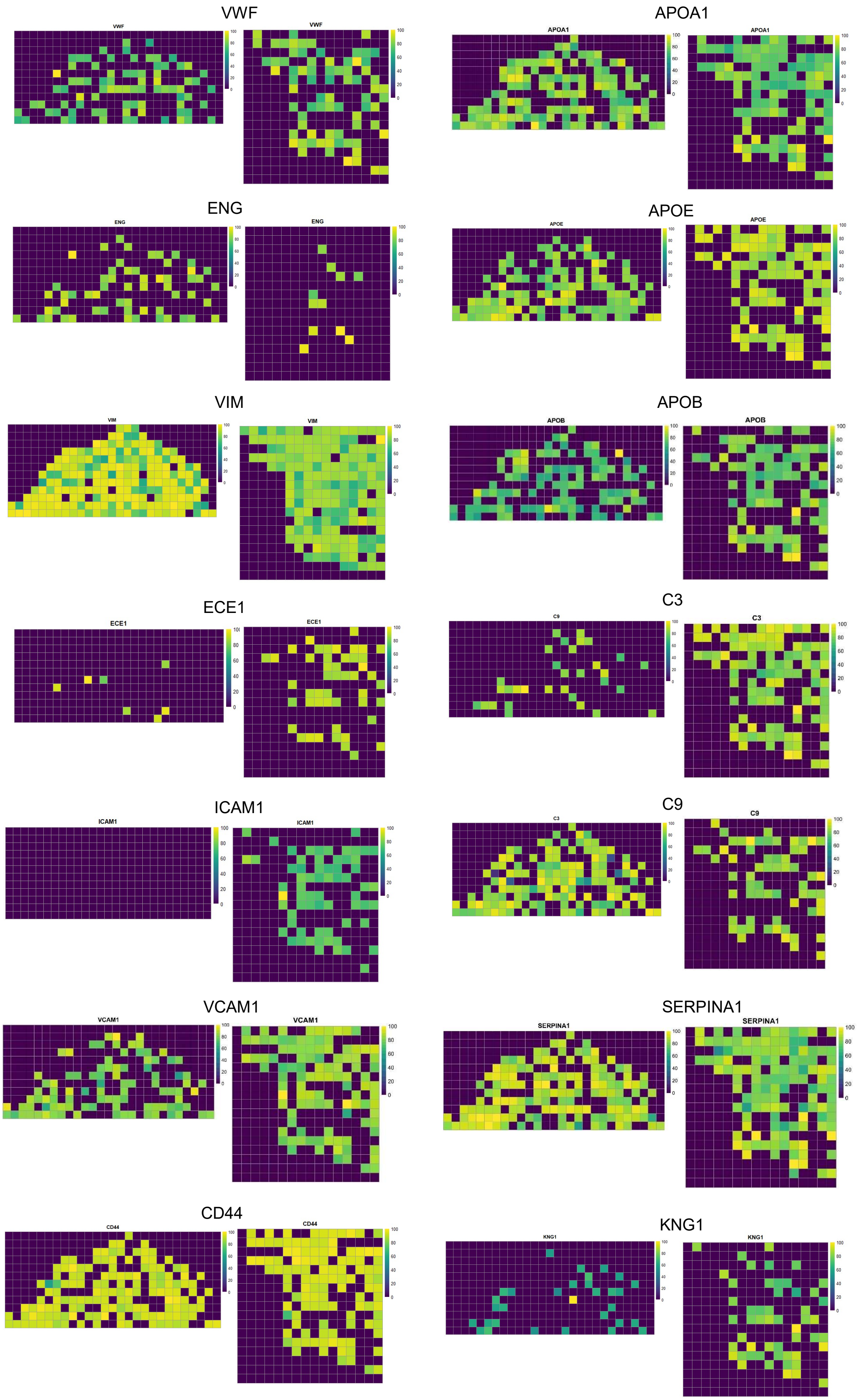

S4b. (continued) Inflammation and angiogenesis markers

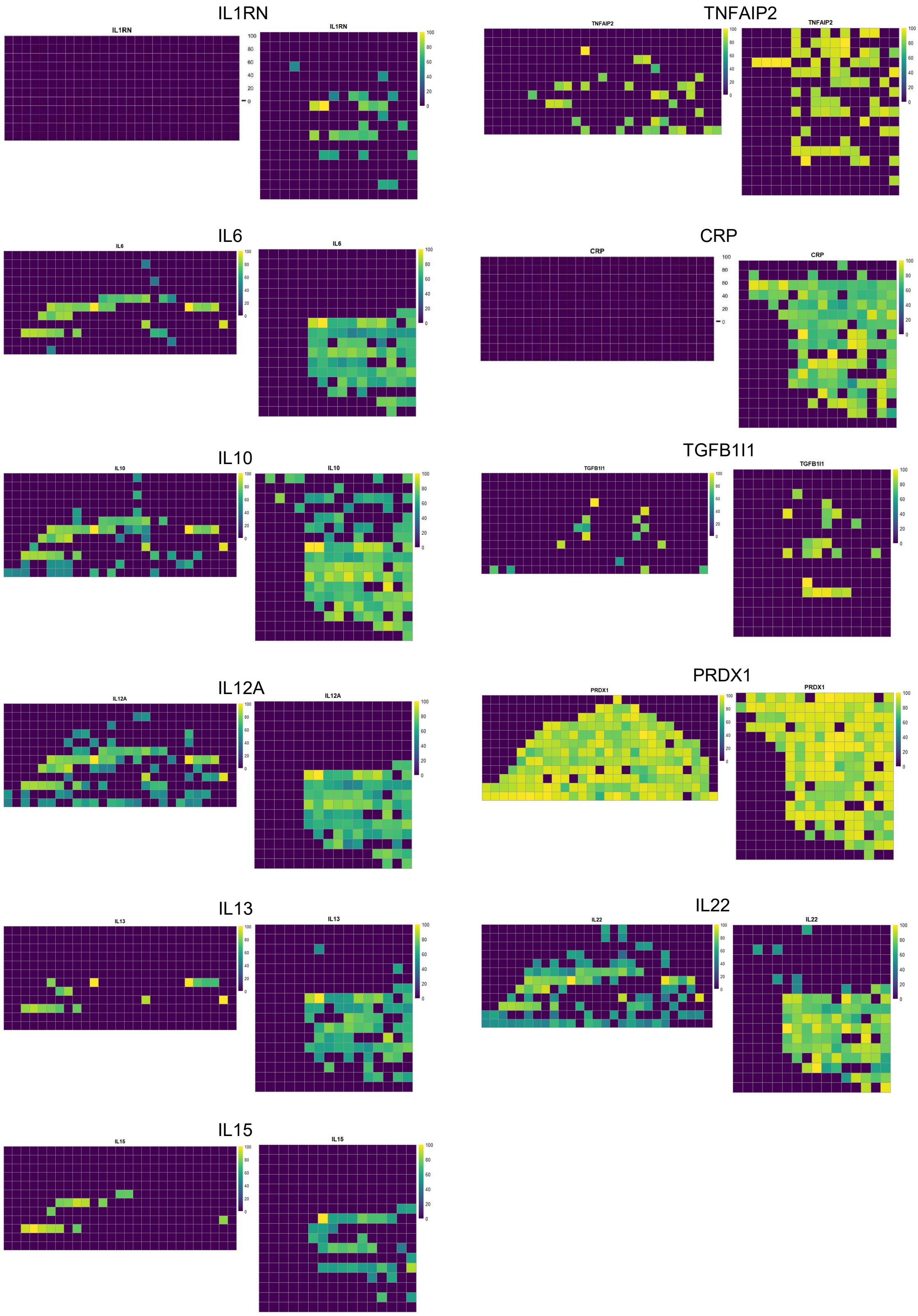

S4c. Synovitis mediators

F2

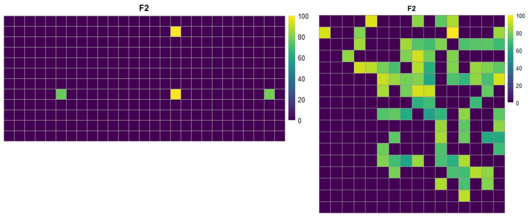

SERPINC1

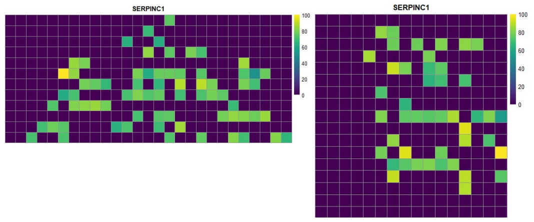

FGG

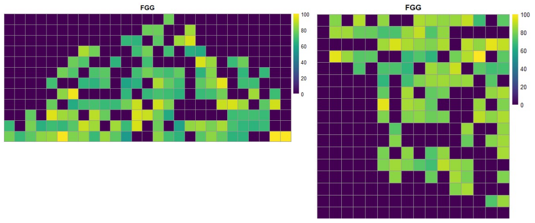

PROCR

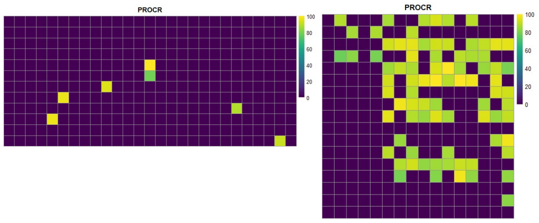

FGA

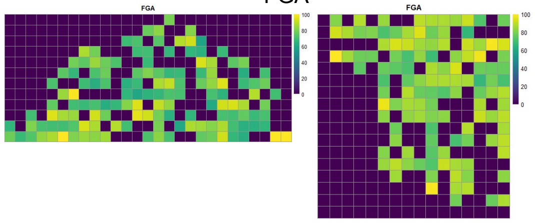

HRG

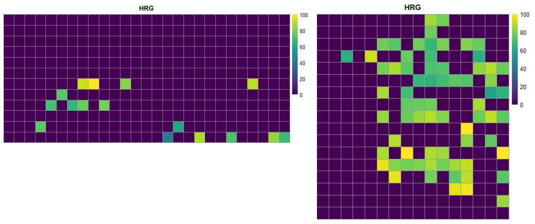

FGB

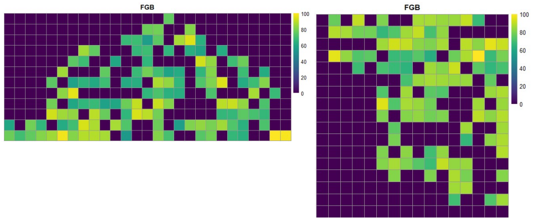

S4d. Metabolic enzyme

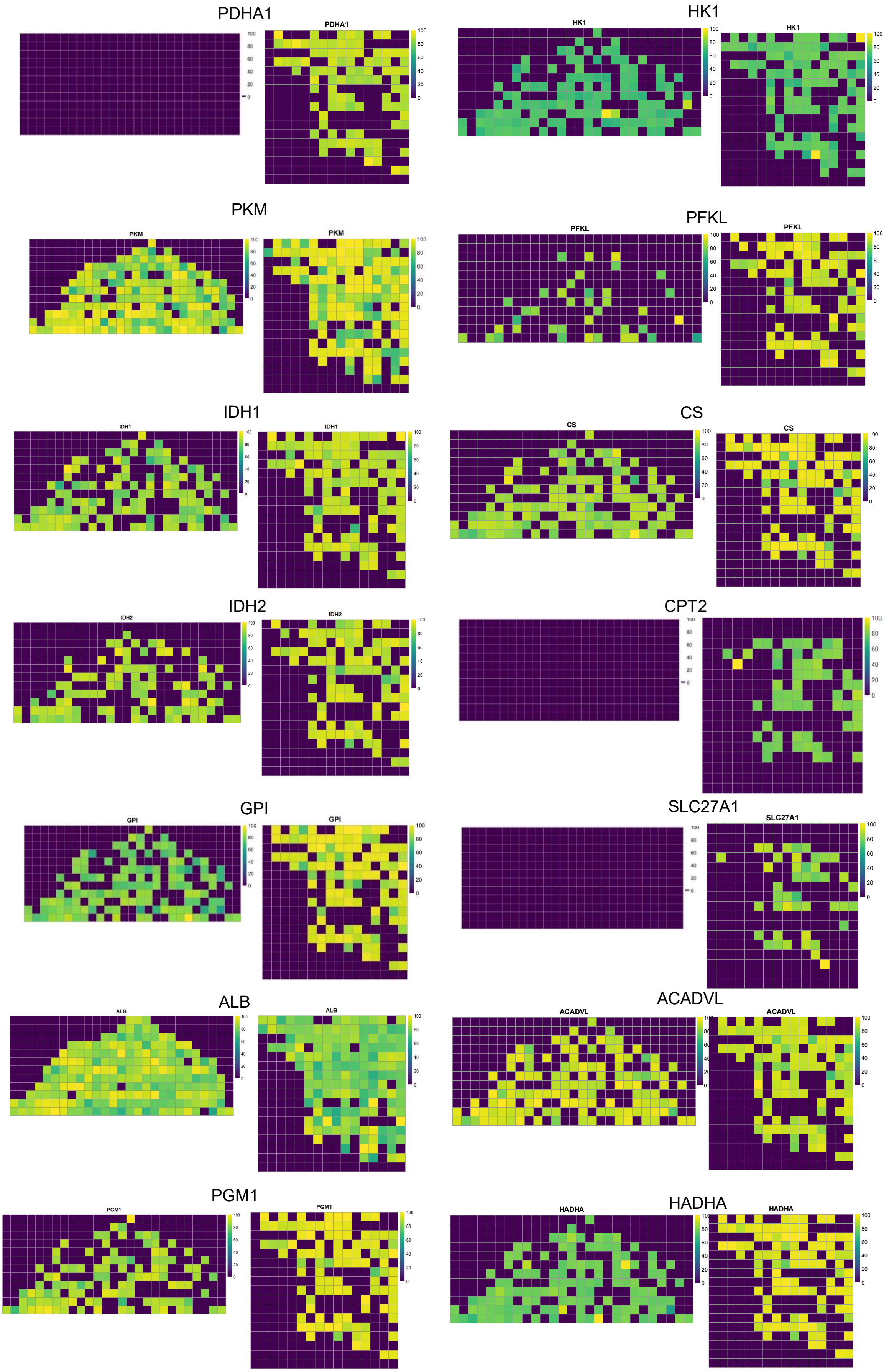

S4e. CD markers

CD9

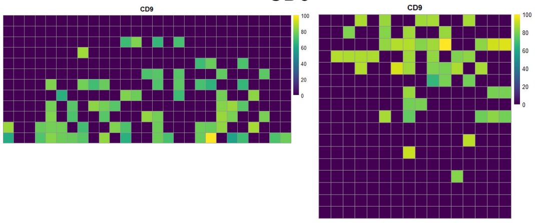

CD99

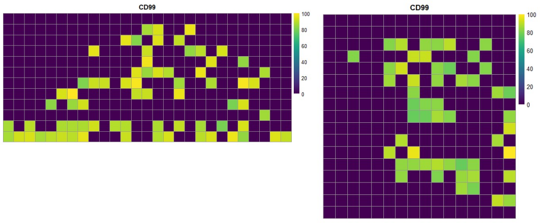

CD163

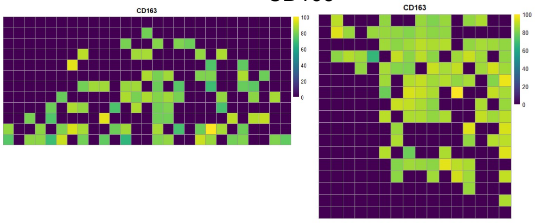

CD36

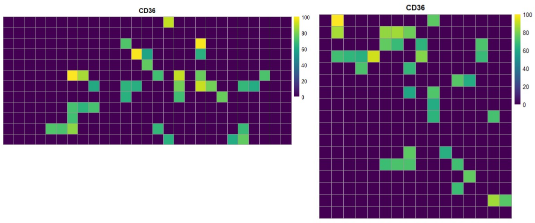

CD109

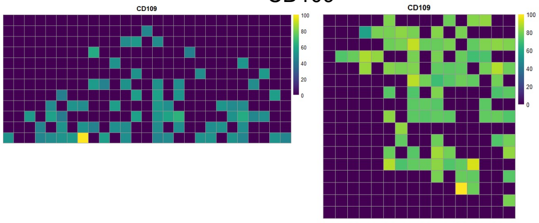

Supplementary Figure S5

a. ECM remodeling, MFA, and fibrosis related markers

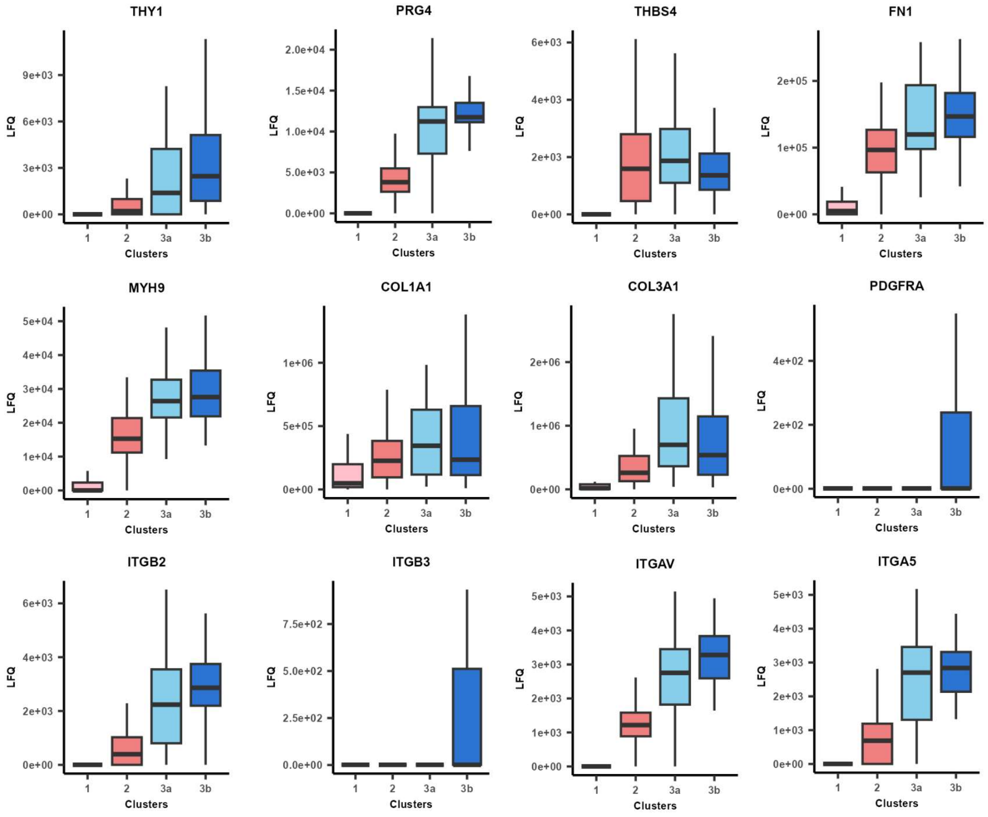

b. CD markers

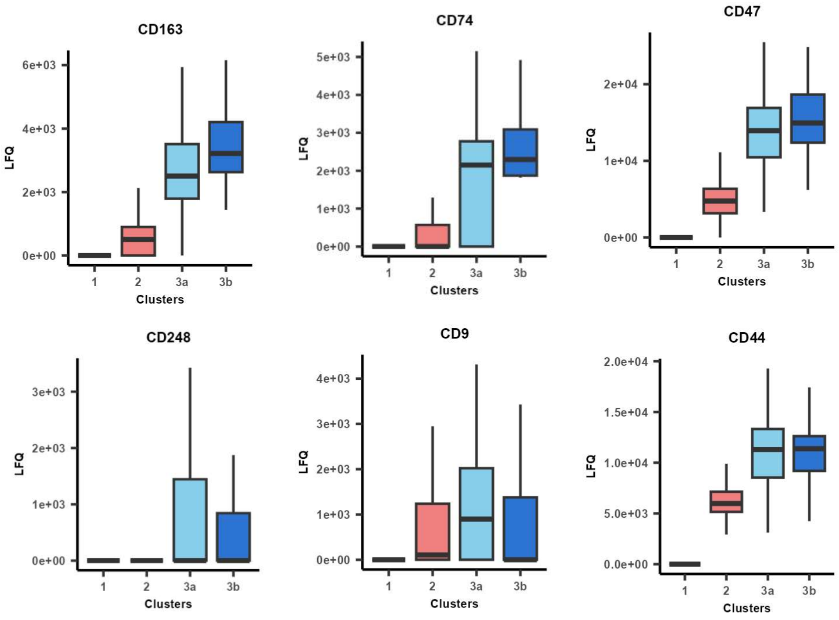

Boxplots of selected functional markers. The median and standard deviation of signal intensities are shown for all voxels within each functional stage.

S5c. Inflammation and angiogenesis markers

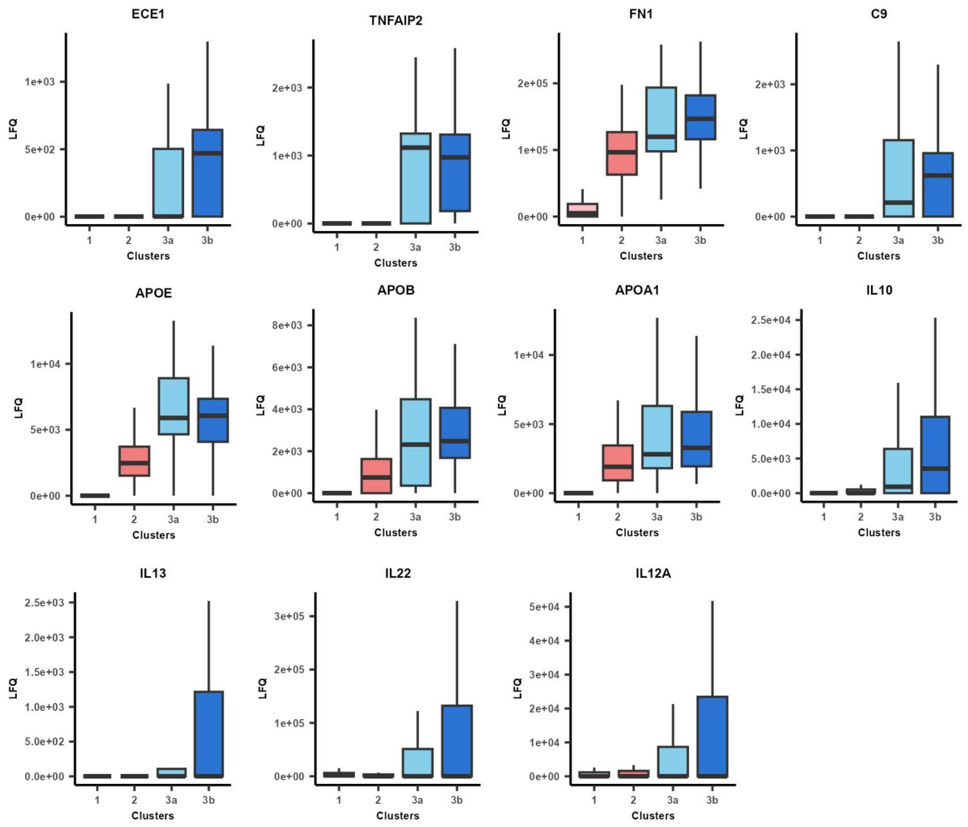

S5d. Metabolically related enzymes

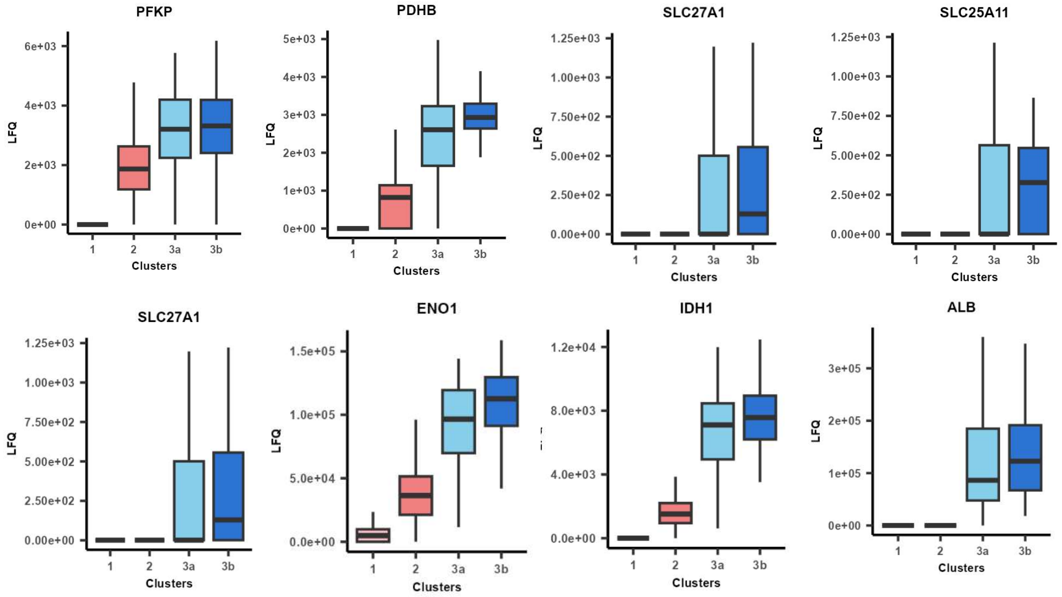

Supplementary Figure S6

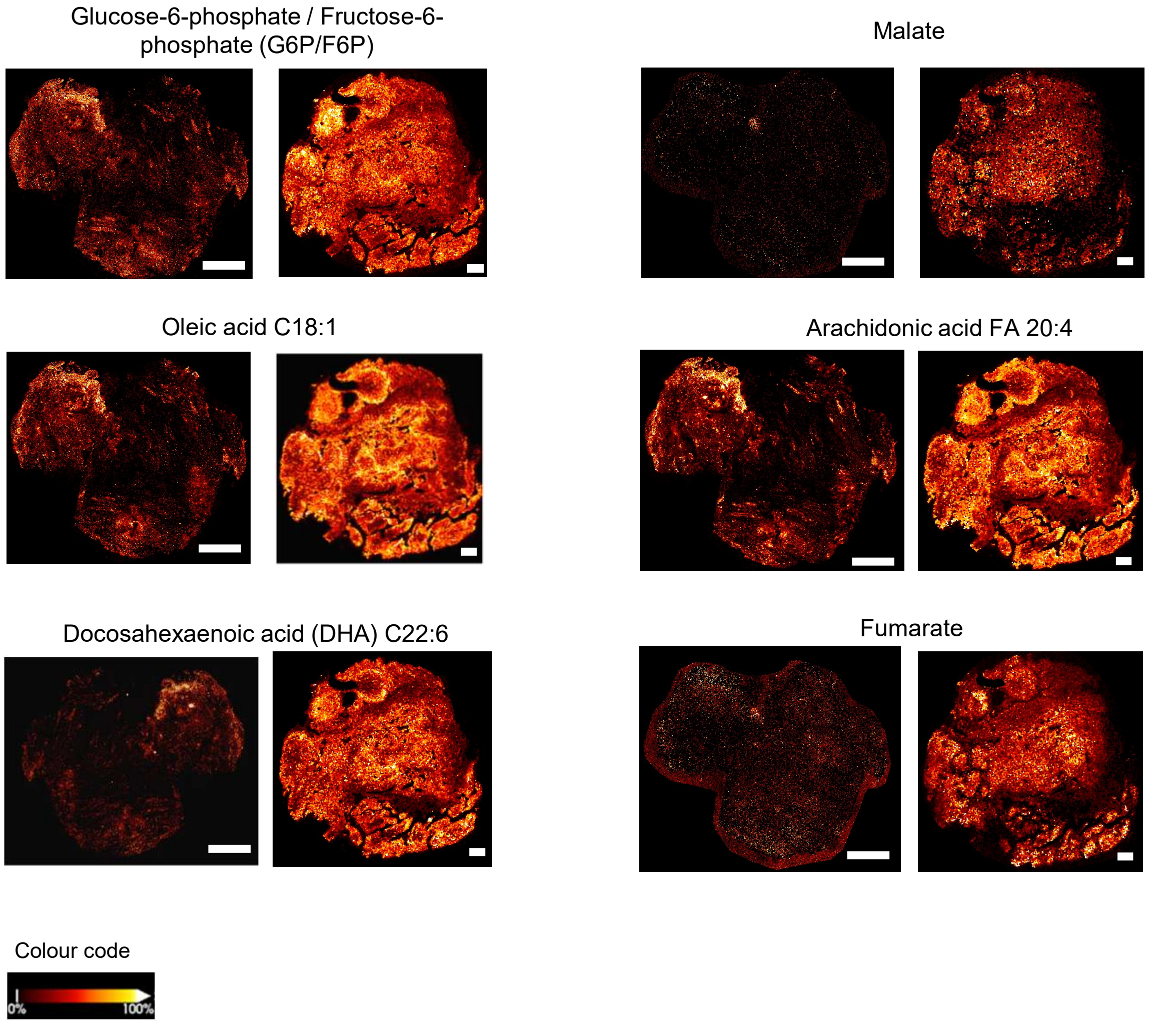

Paired MS images for selected enzyme metabolites. Left: sample 1; right: sample 2. Relative intensity of metabolites post-TIC normalization was shown based on the colour code indicated. Scale bar: 1mm.

Supplementary Figure S7

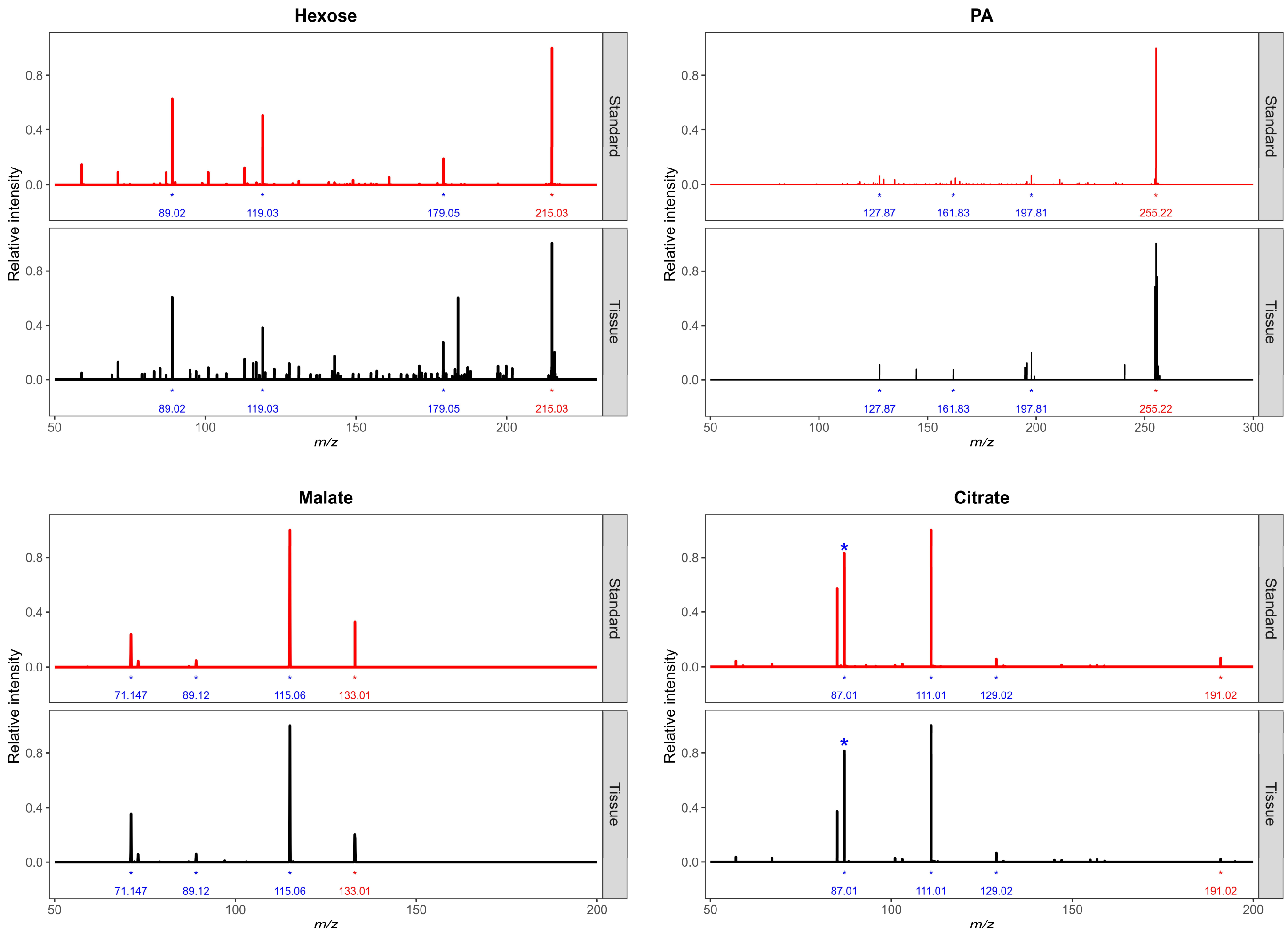

MALDI-TOF-MS/MS spectrum for representative metabolites. For each metabolite, upper panel is the MS2 spectrum generated from metabolite standard; while the bottom panel is the representative spectrum generated from the synovial sample. The * marks the unique fragment ions of the corresponding metabolite for identity confirmation.

Supplementary Figure S8

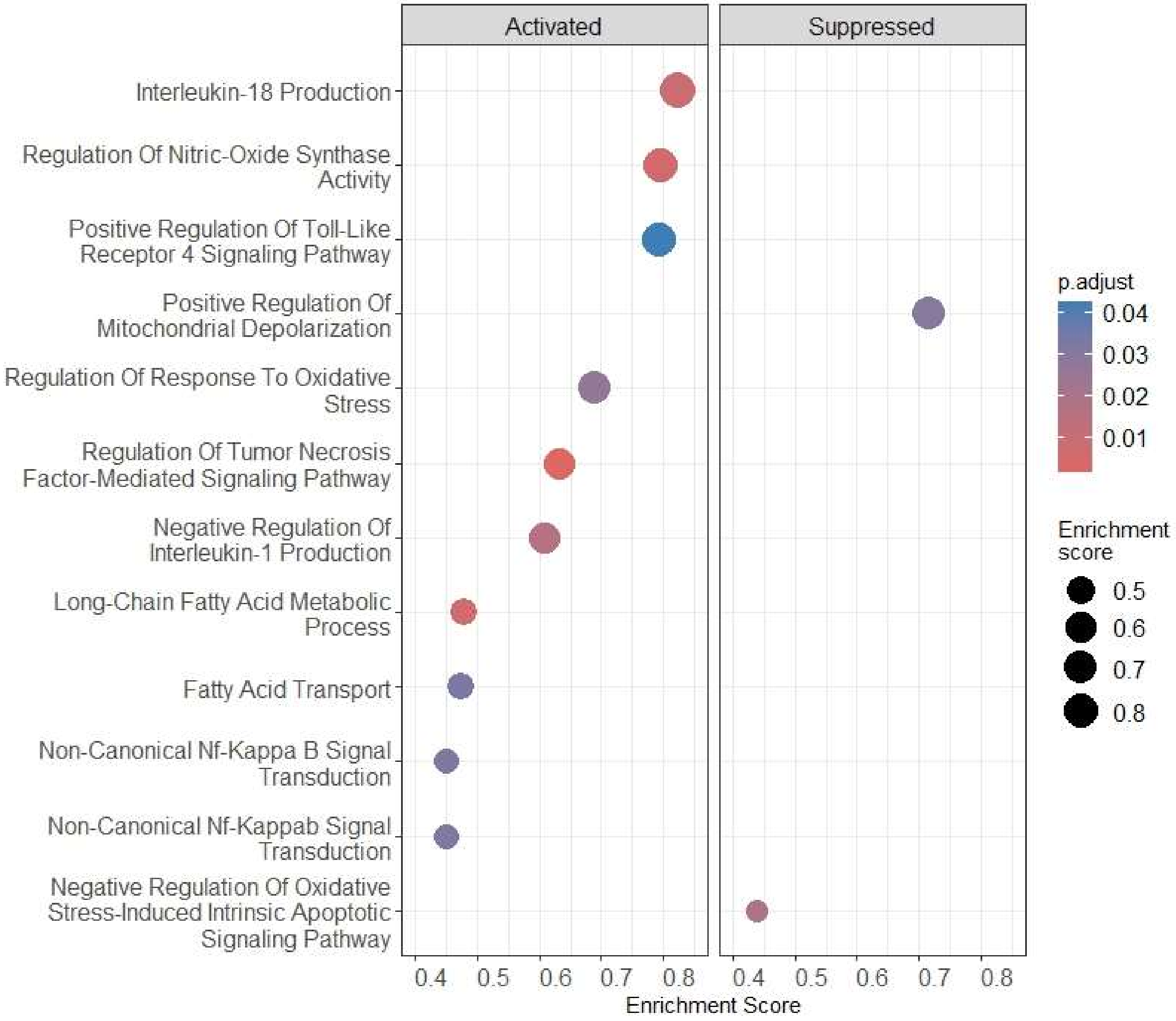

GSEA enrichment based on the quantitation information of proteins among voxel samples between the c3 and c2 regions, respectively. GO/BP terms were used, Benjamini-Hochberg adjusted p-values were applied, and shown in the colour code on the side of the diagram.
